# Style transfer mapping with ensemble learning for cross-subject EEG-based neuropsychiatric disease recognition

**DOI:** 10.3389/fphys.2026.1947840

**Published:** 2026-10-14

**Authors:** Xinmin Ding, Yiming Guo, Shuwei Ge, Shenting Yang, Jipu Su, Minpeng Xu, Xuenan Wang, Hongli Chang

**Affiliations:** 1 Academy of Medical Engineering and Translational Medicine, Tianjin University, Tianjin, China; 2 Institute of Brain Science and Brain-inspired Research, Shandong First Medical University & Shandong Academy of Medical Sciences, Jinan, China; 3 Clinical Medical and Preclinical Medicine School, Shandong First Medical University & Shandong Academy of Medical Sciences, Jinan, China; 4 College of Business, City University of Hong Kong, Hong Kong, Hong Kong SAR, China

**Keywords:** electroencephalogram (EEG), ensemble classifier, neuropsychiatric disease recognition, prototype-consistent style transfer mapping, support vector machine

## Abstract

Cross-subject electroencephalogram (EEG) recognition remains a fundamental challenge because large inter-subject variability causes severe distribution shifts that substantially degrade the generalization ability of existing learning models. Most existing transfer learning methods attempt to align global feature distributions but often overlook subject-specific structural variations, resulting in unstable adaptation under limited calibration data. To address this problem, we propose a Prototype-Consistent Style Transfer Mapping (PC-STM) framework for cross-subject neuropsychiatric disease recognition. Unlike conventional domain adaptation methods, the proposed framework models subject discrepancy as a prototype-guided style transformation that explicitly aligns target representations with class-consistent source prototypes while preserving discriminative structures. Specifically, a multi-source prototype construction strategy is introduced to characterize representative class distributions, followed by a confidence-aware transductive semi-supervised style transfer module that iteratively refines transformation parameters using labeled calibration samples and unlabeled evaluation samples. Furthermore, a reliability-aware ensemble learning strategy dynamically aggregates multiple subject-specific classifiers according to their calibration consistency, thereby improving robustness against heterogeneous subject distributions. Experiments on two public EEG datasets covering major depressive disorder and Parkinson’s disease show stable performance under the evaluated leave-one-pair-out protocol. The calibration labels are inherited from the public dataset cohorts or task conditions, and the study does not constitute prospective clinical diagnostic validation. Beyond the evaluated EEG tasks, prototype-guided style transfer may provide a basis for future investigation of other limited-supervision recognition problems.

## 1 Introduction

Electroencephalogram (EEG) signals provide a non-invasive and high–temporal-resolution approach for characterizing neural activity, and have been widely used in computer-aided recognition of neuropsychiatric disorders such as major depressive disorder (MDD) and Parkinson’s disease (PD) (Sharma et al., 2021; Deng et al., 2022; Tülay, 2024). Despite their advantages, EEG signals exhibit substantial inter-individual heterogeneity arising from anatomical, physiological, and behavioral differences. This variability results in a pronounced distribution shift between subjects, which severely affects the generalizability of conventional classifiers and hinders real-world deployment (Wang et al., 2020; Maitin et al., 2022).

Clinical diagnosis relies on symptoms, medical history, examination, and expert judgment. EEG classifiers are therefore more appropriately described as disease recognition or computer-aided screening systems unless they have undergone prospective diagnostic validation. Computational EEG recognition has nevertheless attracted increasing attention (Li et al., 2019; Peng et al., 2019; Movahed et al., 2021; Saeedi et al., 2021). Existing approaches, such as convolutional neural networks (CNN) (Ruffini et al., 2019), RNN-based architectures (Ruffini et al., 2019), and functional connectivity analysis (Zhang et al., 2020), have achieved promising performance in within-subject or large-sample scenarios. However, their performance often deteriorates sharply when generalized to unseen subjects.

To mitigate cross-subject discrepancies, transfer learning has been widely investigated (Pan and Yang, 2009; Li et al., 2019). Domain alignment methods such as CORAL (Sun and Saenko, 2016), DANN (Ganin et al., 2016), or MMD-based approaches (Long et al., 2015) attempt to reduce distribution discrepancy by learning an invariant feature space (Long et al., 2015). Nevertheless, the high-dimensional, nonlinear, and subject-specific nature of EEG signals limits the effectiveness of these methods, especially when only a small amount of calibration data is available.

As a statistical analogy rather than a claim of clinical decision equivalence, the method draws on case-based reasoning by comparing a new observation with representative prior cases (**Figure 1**). This inspires the idea that cross-subject EEG classification can be improved by mapping a new subject’s EEG style toward previously observed subjects, thereby reducing inter-subject variability.

**Figure 1:**
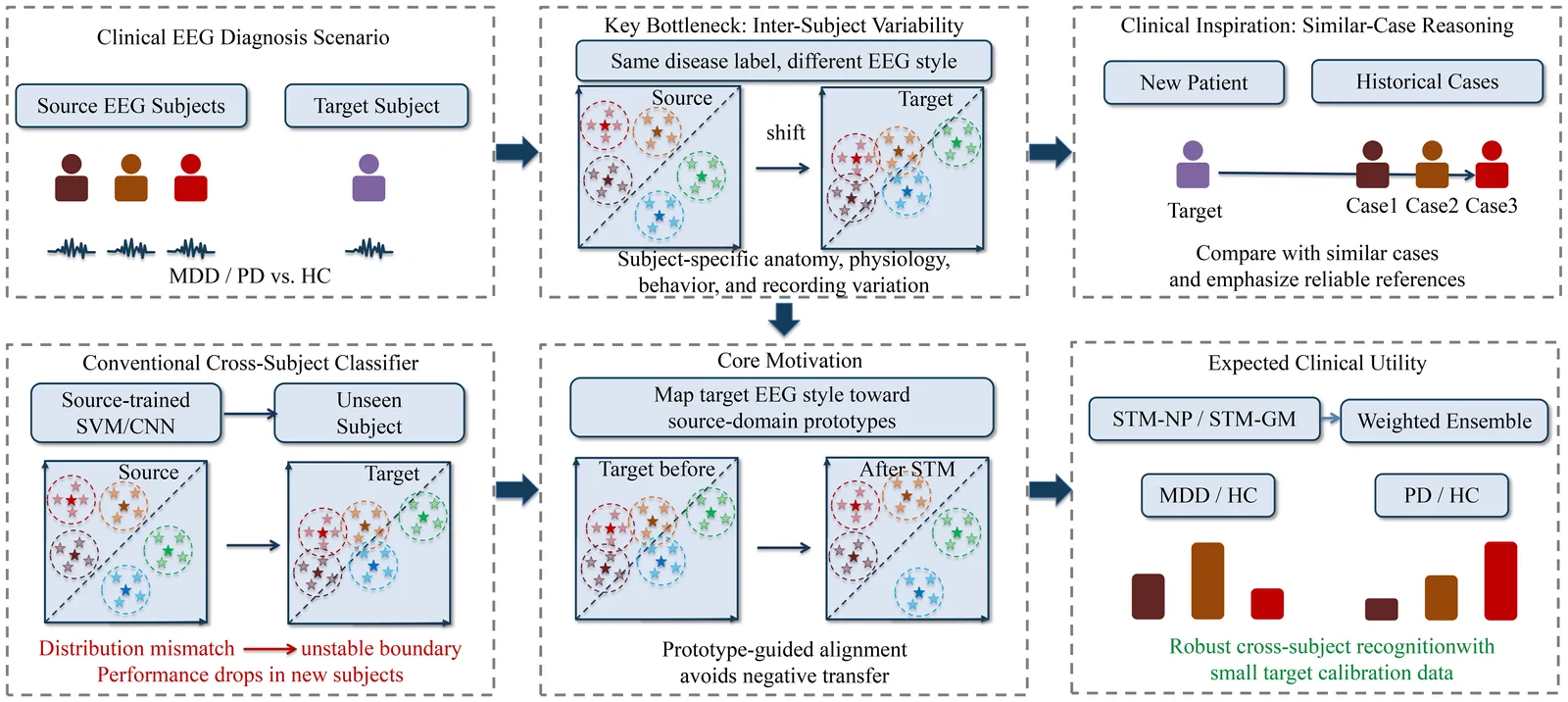
Motivation of prototype-consistent style transfer mapping for cross-subject EEG-based neuropsychiatric disease recognition.

Motivated by this analogy, we introduce PC-STM, which combines a multi-source Style Transfer Mapping module with weighted ensemble classifiers. Hereafter, PC-STM denotes the complete framework, STM denotes only the affine style transfer module, and PC-STM-NP and PC-STM-GM denote the nearest-prototype and Gaussian-model variants, respectively. Unlike prior STM research, this method introduces several new extensions for EEG cross-subject learning: We modify STM into a multi-source, multiprototype framework, constructing class-wise prototypes using k-means and Gaussian modeling (Zhou et al., 2024; Chen et al., 2025). This prevents direct mapping toward individual subjects and mitigates negative transfer. A transductive semisupervised STM is introduced to refine affine mapping parameters using labeled calibration samples and unlabeled evaluation samples, with a confidence model to regulate reliability. We develop a performance-weighted SVM ensemble, where each sub-classifier is trained using a subject pair and weighted based on calibration accuracy. Experiments on two neuropsychiatric disease recognition tasks (MDD and PD), combined with LOPOCV, evaluate the contribution of STM within the PC-STM framework. These contributions together form a statistically motivated framework that combines style alignment with ensemble prediction.

In summary, our work contributes:

1. We formulate cross-subject EEG adaptation as a prototype-consistent style transfer problem and develop a unified style transformation framework that explicitly models subject discrepancy through affine prototype alignment rather than global distribution matching.
2. We introduce a confidence-aware optimization mechanism that jointly exploits labeled calibration samples and unlabeled evaluation samples to iteratively estimate reliable style transformations while suppressing negative transfer.
3. We develop a reliability-aware ensemble inference strategy that dynamically estimates source-domain relevance and integrates heterogeneous classifiers through adaptive confidence weighting.

The proposed framework may also motivate future studies of prototype-guided adaptation in heterogeneous pattern recognition problems beyond EEG.

The remainder of this paper is organized as follows. Section 2 reviews related studies on EEG-based neuropsychiatric disease recognition, cross-subject transfer learning, and prototype-guided style transfer, highlighting the challenges that motivate this work. Section 3 presents the proposed Prototype-Consistent Style Transfer Mapping framework, including EEG feature representation, multi-source prototype construction, confidence-guided style transfer optimization, and reliability-aware ensemble inference. Section 4 provides experimental evaluations on two public EEG datasets, including contextual literature comparisons, component comparisons, and robustness studies under different calibration settings. Section 5 analyzes the findings, practical limitations, and potential extensions. Finally, Section 6 concludes the paper and outlines future research directions.

## 2 Related work

Research on EEG-based neuropsychiatric disease identification has progressed rapidly in recent years. Existing studies can be broadly grouped into three methodological categories (Acharya et al., 2018): EEG feature learning for neurological and psychiatric disorders (Ahmadlou et al., 2012), cross-subject transfer learning and domain adaptation for EEG, and style or prototype-based mapping frameworks (Ahmadlou et al., 2013). This section reviews representative approaches in each direction and highlights the gap addressed by our method.

### 2.1 EEG-based neuropsychiatric disease recognition

A large body of literature has studied EEG markers associated with major depressive disorder (MDD), Parkinson’s disease (PD), and related neuropsychiatric disorders. Early studies primarily relied on bandpower features, nonlinear dynamics, and statistical descriptors (Ahmadlou et al., 2012; Cai et al., 2018a; Movahed et al., 2021). Subsequent advances focused on spatial–temporal representations, including ERP-based biomarkers (Li et al., 2018), functional connectivity metrics (Liu et al., 2020; Movahed et al., 2021), and brain-network-based analyses (Zhang et al., 2018; Zhang et al., 2020).

The emergence of deep learning further promoted EEG-based recognition, enabling automatic feature extraction. CNN- and RNN-based models have demonstrated strong performance in identifying MDD and other affective disorders (Acharya et al., 2018; Ruffini et al., 2019; Saeedi et al., 2021; Liu et al., 2022), while Transformer variants and hybrid architectures were recently proposed to capture global spatiotemporal dependency (Song et al., 2022). Related work has investigated explainable spatiotemporal spectral meta-learning for subject-independent EEG emotion recognition (Zhang et al., 2026), ensemble and temporal convolutional models for auditory attention (Zhao et al., 2024a; Zhao et al., 2024b), and multimodal alignment between brain signals and visual representations (Duan et al., 2025). Similar progress has been made in PD recognition, where CNNs and recurrent models achieved competitive results based on spectral or spectrogram-based features (Ruffini et al., 2019; Oh et al., 2020; Chang et al., 2023a).

Despite these achievements, most methods rely on subject-dependent training or require large amounts of labeled data. When applied to unseen subjects, their performance typically deteriorates significantly due to strong inter-individual EEG variability. This motivates the need for effective cross-subject adaptation mechanisms.

### 2.2 Cross-subject transfer learning and domain adaptation for EEG

Cross-subject transfer learning has emerged as a key strategy to mitigate subject-specific EEG variability. Traditional approaches include domain alignment techniques such as CORAL, which matches second-order statistics between domains, and MMD-based alignment that minimizes distribution discrepancy across feature spaces. Adversarial learning techniques such as DANN and CDAN further attempt to learn domain-invariant features through domain confusion mechanisms.

In EEG research, these methods have shown encouraging results in emotion recognition (Li et al., 2019), motor imagery, and mental workload estimation. However, domain adaptation methods typically assume large training sets or continuous data streams and often require neural network architectures. Their performance becomes unstable when only a small target calibration set is available, which is common in clinical applications. Moreover, adversarial or MMD-based alignment may distort class boundaries when the underlying data distributions are non-Gaussian or multimodal, typical characteristics of neuropsychiatric EEG data.

In contrast, instance-based or prototype-based transfer methods provide a more inspectable adaptation path under small-sample conditions, although procedural transparency does not by itself establish subject-level or neurophysiological interpretability.

Few-shot class-incremental learning provides adjacent methodological context for limited-data adaptation. Recent studies survey classification and object detection (Zhang et al., 2025b) and develop compatible or well-prepared frameworks for pill recognition and retinal disease recognition (Zhang et al., 2025a; Zhang et al., 2025c; Zhang et al., 2026); a survey of neural networks for finger vein recognition likewise discusses representation learning under acquisition variability (Yin et al., 2025). These studies address different prediction tasks and are cited as methodological context rather than direct experimental baselines.

### 2.3 Style transfer mapping and prototype-based alignment

Style transfer mapping (STM) was originally proposed for writer adaptation in handwriting recognition (Zhang and Liu, 2012), where the goal is to align the style of new writers to existing reference domains. STM introduces a regularized affine transformation to reduce stylistic variability and has been shown effective in domains where structural differences between subjects follow linear or locally linear transformations.

The original STM framework supports supervised, unsupervised, and semi-supervised adaptation (Zhang and Liu, 2012), which motivates its extension to calibration-efficient EEG adaptation. However, the use of STM in EEG-based neurological disease recognition remains largely unexplored. EEG signals differ fundamentally from handwriting or vision modalities due to their nonlinear, nonstationary, and highly individualized characteristics. Applying STM to EEG therefore requires modified prototype construction, domain-specific regularization, and robust destination modeling.

Furthermore, ensemble learning has been widely adopted to improve model stability by aggregating multiple classifiers (Hu et al., 2024). Yet, previous methods rarely integrate ensemble strategies with STM-based feature alignment, especially in clinical cross-subject EEG scenarios.

While prior studies have made significant progress in EEG feature learning, domain adaptation, and style-based mapping, there remains an unmet need for a multi-source, calibration-efficient, prototype-driven STM framework specifically designed for cross-subject EEG-based neuropsychiatric disease recognition.

## 3 Methodology

The complete PC-STM framework integrates a multi-source Style Transfer Mapping (STM) module with a performance-weighted ensemble classifier to address cross-subject EEG variability. As illustrated in **Figure 2**, the method operates in four stages: feature extraction, ensemble construction, style transfer mapping, and classification. Prototype construction precedes supervised STM initialization, which is followed by transductive refinement and weighted ensemble prediction. This section introduces the data representation, weighted SVM ensemble, multi-source STM, and confidence mechanism.

**Figure 2:**
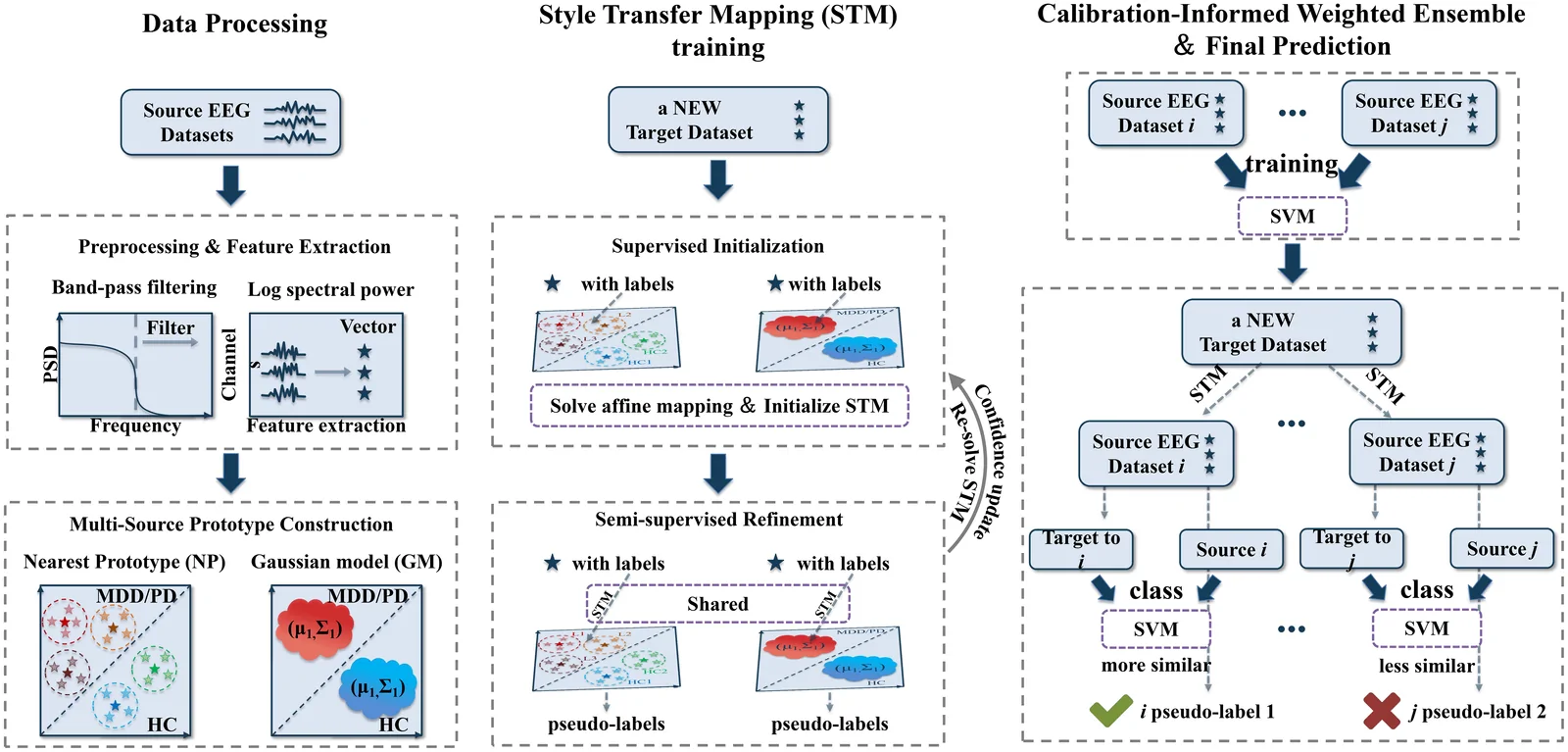
The proposed neuropsychiatric disease recognition framework.

As illustrated in **Figure 2**, this framework comprises several core modules and operates in four main stages. First, preprocessed EEG data from multiple subject pairs are used to train individual SVM subclassifiers, each corresponding to a specific subject pair. These sub-classifiers are evaluated based on their performance on calibration data and integrated into a weighted ensemble classifier. Second, target data are partitioned into labeled calibration trials and unlabeled evaluation trials. The labeled data are used to learn the transformation parameters of the STM module. Third, STM aligns the evaluation samples to the source domain by minimizing a weighted least squares error. This alignment is performed via nearest-prototype matching or a Gaussian model-based projection. Finally, the transformed evaluation samples are classified using the ensemble classifier, which combines sub-classifier outputs according to their weights. As a computational analogy to case-based reasoning, PC-STM addresses individual variability in retrospective EEG disease recognition.

### 3.1 Data and preprocessing

In this study, we employed the MODMA dataset ^1^, which encompasses 128-channel Event-Related Potentials (ERPs) collected from 24 individuals diagnosed with MDD and 29 healthy control participants. The ages of the subjects ranged from 16 to 52 years. Data acquisition was conducted at a sampling frequency of 250 Hz. It involved a dot-probe task that presented pairs of faces categorized as happy-neutral (happy cue), fearful-neutral (fear cue), and sad-neutral (sad cue). Adhering to the principles of gender and age matching, we selected a total of 42 participants from the MODMA dataset, organizing them into 21 matched pairs. Comprehensive details regarding the dataset are provided in **Table 1**, where ‘F’ denotes female and ‘M’ denotes male participants. The table reveals a total of 21 groups, comprising 12 male groups and 9 female groups. The overall mean age of the participants is 31.40 years, with an age range of 16 to 52 years. Notably, the average age for female participants is slightly lower at 29.17 years, compared to an average age of 33.08 years for male participants. Furthermore, the analysis of the paired subjects indicates an average age difference of merely 1.38 years, reflecting a minimal age disparity within the pairs. Within each happy, fear, or sad cue condition, the prediction target is MDD versus healthy control; the cue is not part of the class label. The All condition pools trials from the three cue conditions while retaining the same disease-group target. Calibration labels are inherited from the participant’s dataset cohort rather than newly assigned trial-level clinical diagnoses.

**Table 1:** Detailed list of subject pairing settings for depression and healthy controls in STM experiment.. (MDD, major depressive disorder; HC, healthy control; F, female; M, male.).

No.	MDD				HC	
	Subject ID	Age	Gender	Subject ID	Age	Gender
1	02010004	25	F	02030005	23	F
2	02010005	20	M	02020008	20	M
3	02010006	42	M	02020016	40	M
4	02010008	42	M	02020022	41	M
5	02010010	34	M	02020018	34	M
6	02010013	21	F	02030007	22	F
7	02010015	26	M	02020013	27	M
8	02010016	28	F	02020014	29	F
9	02010018	30	M	02020019	31	M
10	02010019	24	F	02030006	23	F
11	02010021	45	F	02030020	41	F
12	02010022	52	M	02030017	52	M
13	02010023	42	F	02020015	41	F
14	02010024	20	F	02030004	22	F
15	02010025	28	M	02030019	28	M
16	02010026	16	M	02020026	19	M
17	02010028	29	M	02020020	29	M
18	02010030	35	F	02030014	34	F
19	02010033	24	F	02030021	26	F
20	02010034	28	M	02030018	29	M
21	02010036	46	M	02020010	51	M

The EEGLAB toolbox ^2^ was employed to preprocess the original dataset. We extracted log spectral power features from each electrode across five distinct frequency bands: 1–8 Hz (delta plus theta), 8–12 Hz (alpha), 12–35 Hz (beta), 35–70 Hz (gamma-1), and 70–100 Hz (gamma-2). This extraction process involved filtering the EEG signals using a non-overlapping 0.5-second windowed order-8 zero-phase IIR Butterworth filter, followed by the computation of the logarithm of the root mean square for each window (Chang et al., 2023a).

The Parkinson’s Disease (PD) database^3^, which includes a healthy control group, a PD ON group, and a PD OFF group (Cavanagh et al., 2018), is detailed in **Figure 3**. The data was organized into two categories: the source and target domains. The source domain, which simulates a multi-subject database, consists of labeled EEG data from individuals diagnosed with PD as well as from healthy control participants, ensuring that these groups are matched in terms of gender, age, and sample size. This domain serves as the primary training dataset for the classifier. Conversely, the target domain encompasses the EEG data from test subjects, from which a small subset of samples was randomly selected and labeled to function as calibration data for the style transfer mapping process. For PD, the prediction target is PD ON versus healthy control or PD OFF versus healthy control. The calibration labels are inherited from the dataset diagnosis and medication-state grouping and are not newly assigned by a clinician for individual trials. To analyze the data, we extracted log spectral power features from each electrode across five frequency bands: delta (0.1–3 Hz), theta (4–7 Hz), alpha (8–11 Hz), beta (12–29 Hz), and gamma (30–49 Hz). This was accomplished by applying an order-8 zero-phase IIR Butterworth filter to the EEG signals within these frequency bands, followed by the computation of the logarithm of the root-mean-square values (Chang et al., 2023a).

**Figure 3:**
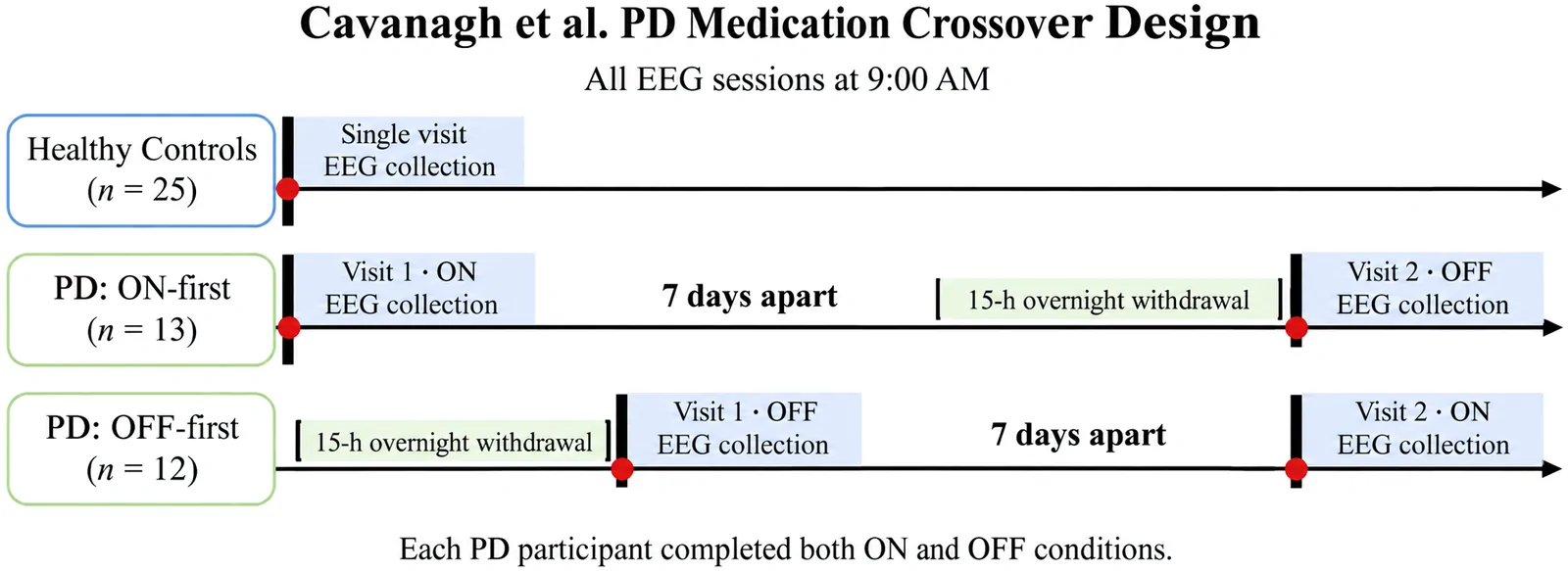
Experimental data collection conditions and time.

### 3.2 Evaluation protocol and target data use

All experiments use leave-one-pair-out cross-validation. In each fold, one matched patient-control pair is held out, and the held-out pair is excluded from source prototype construction, source SVM training, and source-domain parameter selection. Labeled trials from the held-out pair form the calibration subset, while all remaining trials form the unlabeled evaluation subset and their ground-truth labels remain hidden until performance is calculated. Because the complete unlabeled evaluation subset participates in STM refinement through pseudo-labels and confidence weights, the protocol is transductive semi-supervised adaptation rather than purely inductive cross-subject classification. Pair-based evaluation was selected to control age and gender differences, but it is distinct from conventional leave-one-subject-out validation and should be interpreted within this matched design.

### 3.3 EEG feature representation

Given an EEG trial $x\in {\mathbb{R}}^{C\times T}$ with *C* channels and *T* time points, the signal is filtered into five canonical frequency bands. For each band, the root-mean-square (RMS) power is computed and log-transformed. A feature vector is formed as $o\in {\mathbb{R}}^{C\times 5}$. These features constitute the input to both STM and the ensemble classifier.

### 3.4 Cross-subject distribution alignment

A fundamental challenge in cross-subject EEG recognition is the distribution discrepancy between source and target subjects caused by anatomical variability, physiological differences, recording conditions, and individual behavioral patterns. Let the source-domain EEG features and target-domain EEG features be sampled from two different probability distributions specified in Equation 1:

(1) $$x_{s}∼P_{s}(x),\;x_{t}∼P_{t}(x),$$

where $P_{s}(x)$ and $P_{t}(x)$ denote the source and target feature distributions, respectively.

Most existing domain adaptation methods aim to learn a feature transformation *f*(·) that satisfies the distribution-alignment relation in Equation 2:

(2) $$P_{s}(f(x))\approx P_{t}(f(x)),$$

by minimizing a global distribution discrepancy, e.g., Maximum Mean Discrepancy (MMD), covariance alignment, or adversarial objectives. Although effective for many computer vision tasks, these approaches often ignore the intrinsic class structure and may distort decision boundaries when the target distribution exhibits multimodal characteristics, which is common in EEG signals due to strong inter-subject variability.

Instead of directly aligning the global feature distributions, the proposed framework formulates cross-subject adaptation as a prototype-guided style transfer problem. Specifically, an affine transformation in Equation 3:

(3) T(x)=Ax+b

is learned to map target EEG features toward the class-conditioned prototype space. Consequently, the optimization objective can be expressed in Equation 4:

(4) $$\underset{A,b}{\min}\sum_{i=1}^{N}∥Ax_{i}+b-p_{y_{i}}{∥}_{2}^{2},$$

where $p_{y_{i}}$ denotes the prototype corresponding to the class label *y_i_*.

Unlike conventional domain adaptation methods that enforce global distribution consistency, the proposed formulation explicitly preserves class-specific structures while removing subject-dependent style variations. Therefore, the learned transformation simultaneously reduces inter-subject discrepancy and maintains discriminative information required for reliable classification.

### 3.5 Prototype variance analysis

The proposed framework aligns target samples with class prototypes rather than individual source samples. For the *c*-th class, the prototype is defined by Equation 5:

(5) $$p_{c}=\frac{1}{N_{c}}\sum_{i:y_{i}=c}x_{i},$$

where *N_c_* is the number of samples belonging to class *c*.

Assuming that samples are independently drawn from the same distribution with covariance matrix Σ, the covariance of the prototype is given by Equation 6:

(6) $$\text{Cov}(p_{c})=\frac{1}{N_{c}}\text{Σ}.$$

The corresponding variance relation is given in Equation 7:

(7) $$\text{Var}(p_{c})=\frac{1}{N_{c}}\text{Var}(x),$$

which indicates that the prototype provides a significantly lower-variance estimate than individual observations.

Consequently, learning the affine transformation based on prototype supervision reduces estimation variance and improves robustness under limited calibration samples. This property also explains the superior cross-subject generalization observed in the experimental results.

### 3.6 Weighted SVM ensemble classifier

Let $\;S=\{S_{1},S_{2},\ldots ,S_{N}\}$ denote N source subject pairs used for classifier training. For each pair $S_{i}$, an SVM sub-classifier $f_{i}(\cdot )$ is trained using its subject-specific samples.

During testing, a small labeled calibration set $D_{\text{cal}}$ from the target subject is used to measure the reliability of each sub-classifier. The weight of *f_i_* is computed as:


$$w_{i}=\frac{\text{Acc}(f_{i},D_{\text{cal}})}{\sum_{j=1}^{N}\text{Acc}(f_{j},D_{\text{cal}})}.$$

The ensemble decision output is then given by:


$$F(x)=\sum_{i=1}^{N}w_{i}f_{i}(x)$$

This formulation ensures that classifiers trained on subjects more similar to the target subject receive higher influence.

### 3.7 Multi-source style transfer mapping

#### 3.7.1 Affine mapping formulation

To reduce inter-subject distribution shifts, STM learns an affine transformation mapping each target feature *o_i_* to a destination prototype *d_i_'*:


di'=Aoi+b, A∈ℝm×m, b∈ℝm.

The parameters (*A,b*) are obtained by minimizing a regularized weighted least-squares objective in Equation 8:

(8) $$\underset{A,b}{\min}\sum_{i=1}^{n}f_{i}\text{||}Ao_{i}+b-d_{i}{\text{||}}_{2}^{2}+\beta \text{||}A-I{\text{||}}_{F}^{2}+\gamma \text{||}b{\text{||}}_{2}^{2},$$

where *I* is the identity matrix, *f_i_* is a confidence value, and (*β,γ*) control deviation from the identity transformation.

The analytic solution follows:


$$A=QP^{-1},\;b=\frac{1}{f^{\prime }}(d^{\prime }-Ao^{\prime }),$$

where:


$$Q=\sum_{i=1}^{n}f_{i}d_{i}o_{i}^{⊤}-\frac{1}{f^{\prime }}d^{\prime }o^{\prime }⊤+\beta I,$$

$$P=\sum_{i=1}^{n}f_{i}o_{i}o_{i}^{⊤}-\frac{1}{f^{\prime }}o^{\prime }o^{\prime }⊤+\beta I,$$

and:


$$o^{\prime }=\sum_{i=1}^{n}f_{i}o_{i},\;d^{\prime }=\sum_{i=1}^{n}f_{i}d_{i},\;f^{\prime }=\sum_{i=1}^{n}f_{i}+\gamma .$$

#### 3.7.2 Prototype-based destination construction

To avoid negative transfer, each target sample is not mapped to specific source samples but to class-wise prototypes.

##### 3.7.2.1 Nearest prototype

Let P denote the prototype set. The destination is assigned as:


$$d_{i}=\text{arg }\underset{p\in P}{\min}\text{||}o_{i}-p{\text{||}}_{2}.$$

##### 3.7.2.2 Gaussian model

Alternatively, a Gaussian-based projection accounts for class covariance:


$$P(o_{i})=\mu +\text{min }(1,\frac{\rho }{d(o_{i})})(o_{i}-\mu ),$$

where *d*(*o_i_*) is the Mahalanobis distance and *µ* is the class mean. GM preserves geometric distribution and provides smoother transformations.

### 3.8 Transductive semi-supervised STM with confidence weighting

After supervised initialization with the labeled calibration subset, STM uses the complete unlabeled evaluation subset for transductive semi-supervised refinement. Predicted pseudo-labels and confidence weights, rather than hidden ground-truth labels, determine the contribution of these samples. Confidence for each sample is computed as:


$$F(o_{i})=\sigma (\theta (d_{2}-d_{1})+\tau ),$$

where *d*_1_ and *d*_2_ denote distances to the nearest and second-nearest prototypes, and *σ*(·) is the logistic function. High-confidence samples contribute more strongly to the mapping objective (Cai et al., 2018b), improving mapping robustness.

The final STM parameters (*A,b*) are applied to transform all target EEG features, which are then classified by the weighted ensemble. **Algorithm 1** summarizes the proposed PC-STM framework.

Algorithm 1

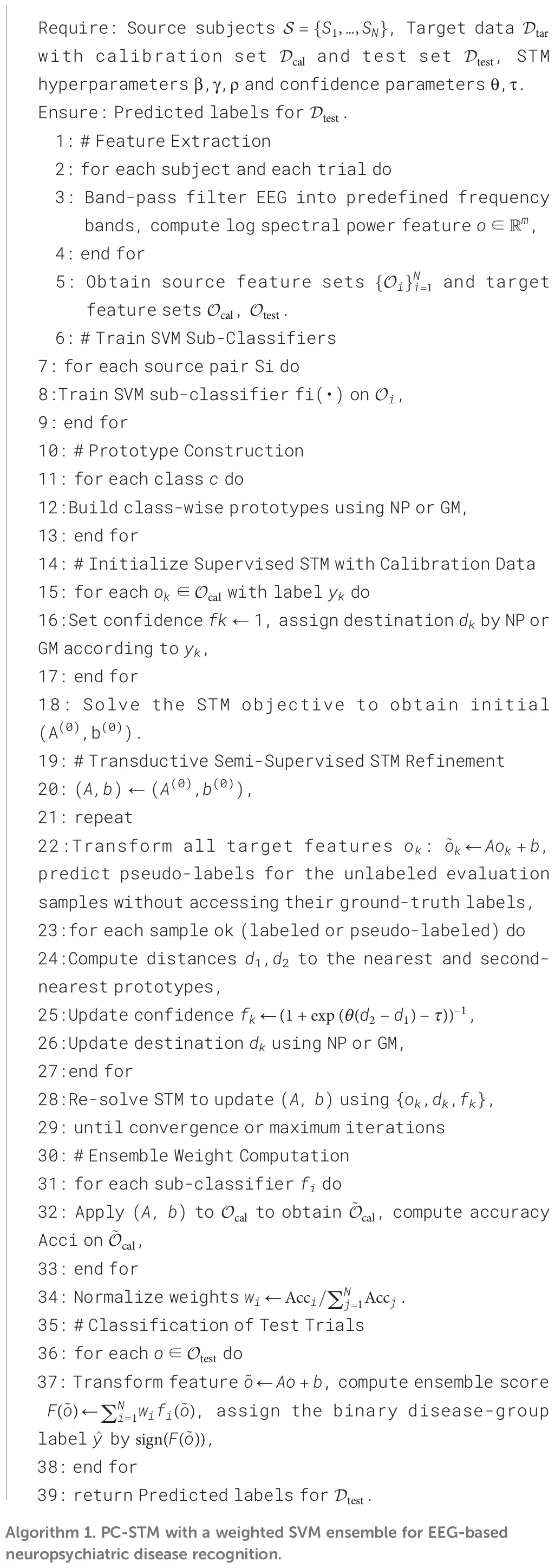

## 4 Results

This section reports the experimental evaluation of PC-STM on two neuropsychiatric disease recognition tasks: major depressive disorder (MDD) and Parkinson’s disease (PD). All experiments adopt the leave-one-pair-out cross-validation (LOPOCV) protocol to preserve matched patient-control evaluation (Chang et al., 2023a). The held-out pair is completely excluded from source prototype construction, source SVM training, and source-domain parameter selection. We compare three configurations: (1) Ensemble without STM, (2) PC-STM-NP using nearest-prototype destinations, and (3) PC-STM-GM using Gaussian-model destinations. These comparisons assess the contribution of the STM module and destination construction, but they do not constitute an independent ablation of every internal component.

### 4.1 MDD recognition results

**Figure 4** illustrates recognition accuracies across 21 subject pairs and four reporting conditions (*Happy*, *Fear*, *Sad*, and *All*). Happy, Fear, and Sad each evaluate MDD versus healthy control within the corresponding cue condition; All pools trials from the three cue conditions while retaining MDD versus healthy control as the prediction target. Across all conditions, both PC-STM variants outperform the Ensemble without STM configuration. In particular, PC-STM-GM achieves the highest stability, with accuracies frequently approaching 100%.

**Figure 4:**
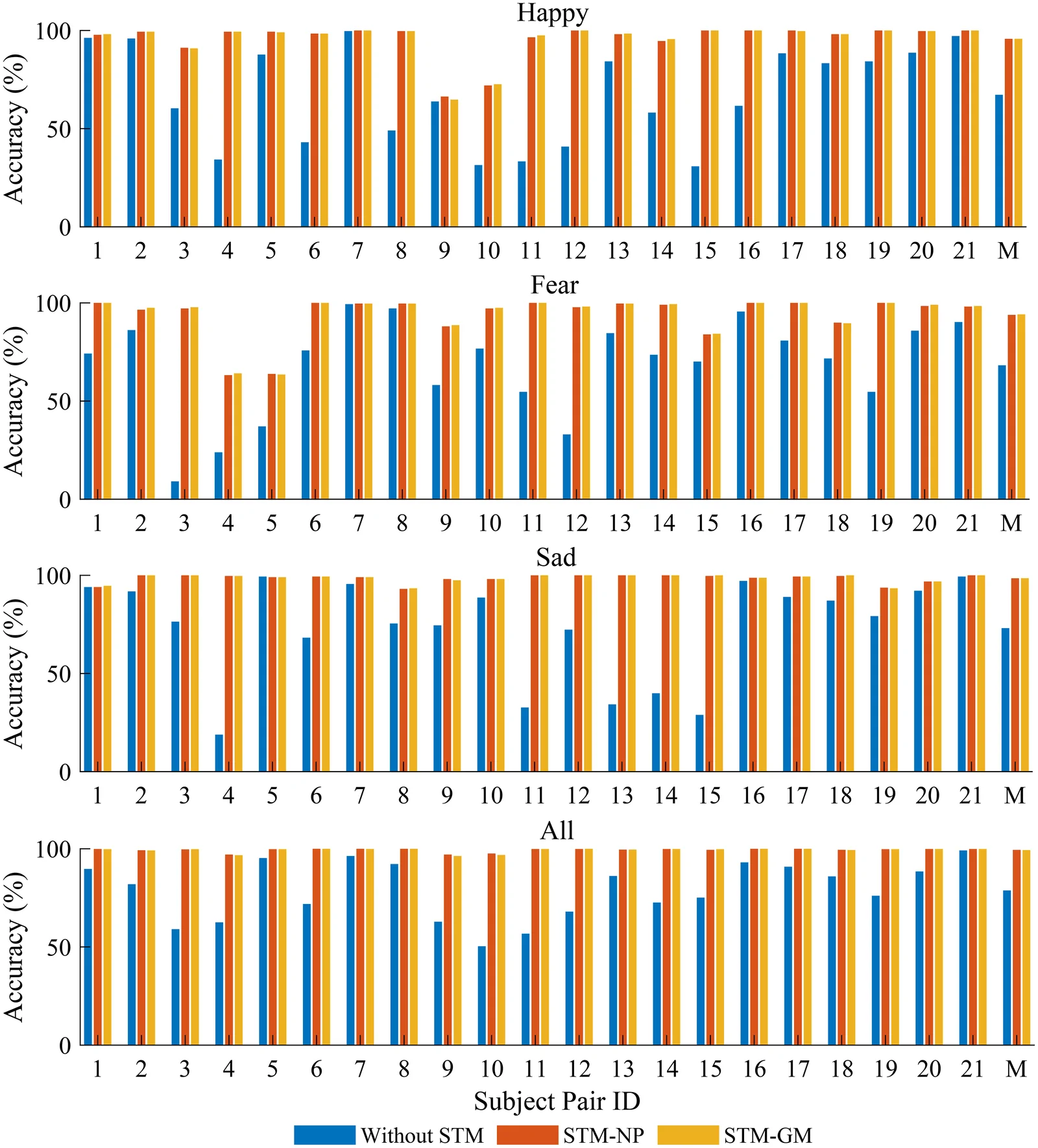
Accuracy of the three methods in the MDD experiment. The label “M” on the x-axis denotes the mean accuracy across all 21 subjects.

The baseline method exhibits strong variability and comparatively low accuracy, especially in the *Fear* and *Sad* cues. In contrast, PC-STM-NP and PC-STM-GM reduce this variability by aligning target subjects to source prototypes before classification.

**Figure 5** presents the confusion matrices for the four reporting conditions. The Ensemble without STM approach yields notable misclassification rates, particularly for MDD samples under the *Fear* and *Sad* cue conditions. Conversely, PC-STM-NP and PC-STM-GM achieve above 93% accuracy for all four conditions. PC-STM-GM has misclassification rates below 2% in the *All* condition.

**Figure 5:**
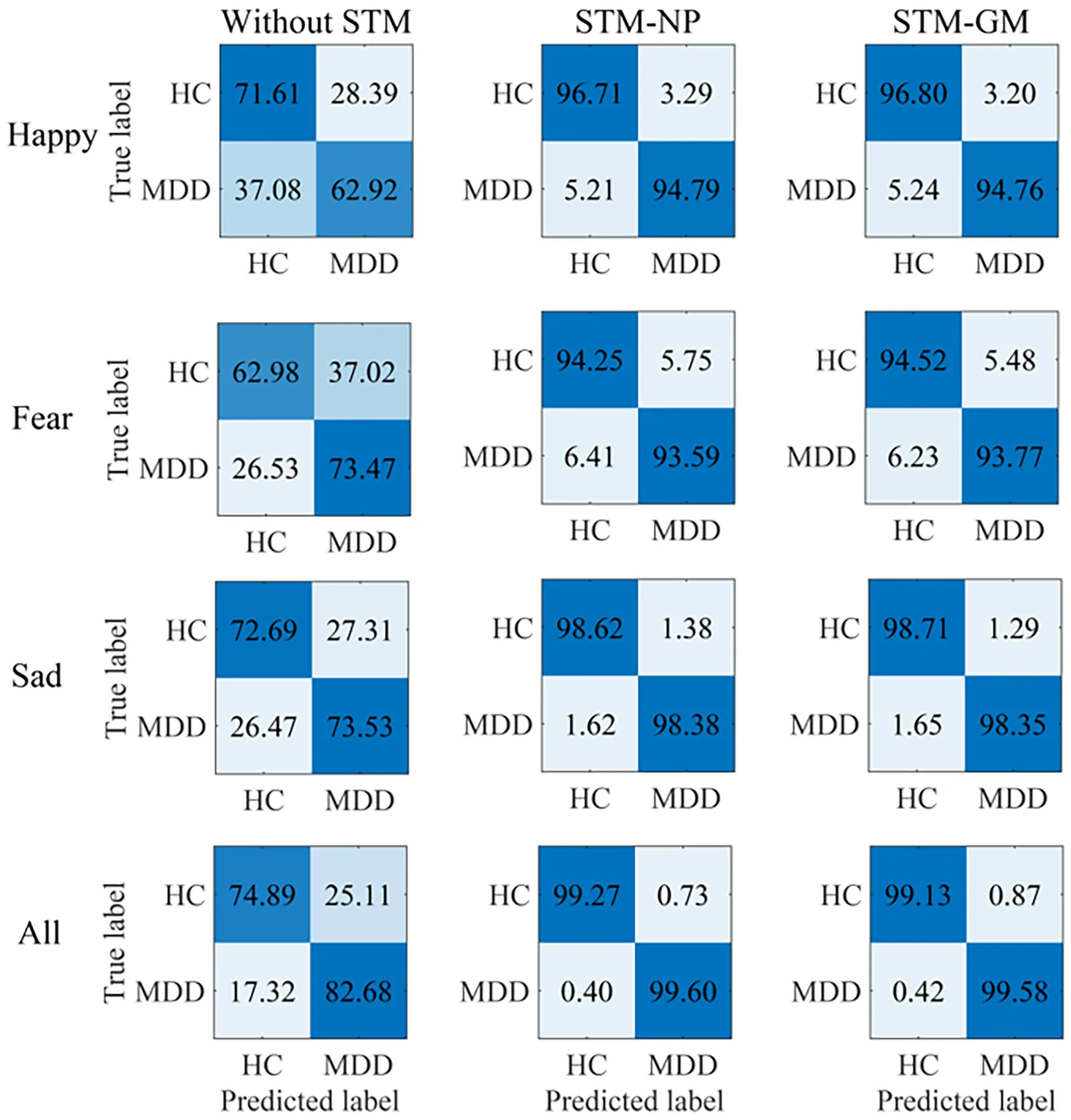
Confusion matrix of the three methods in the MDD experiment.

A summary comparison with previously reported MDD recognition methods is provided in **Table 2**. These values provide contextual references rather than controlled head-to-head comparisons because the studies use different cohorts, channel numbers, preprocessing methods, features, and validation protocols.

**Table 2:** Contextual comparison of EEG depression recognition results reported under different experimental protocols.

Method	Subject	Channel	Feature	Protocol	Accuracy
	(MDD, HC)				(%)
Independent component analysis (Zhang et al., 2018)	(13, 13)	64	–	–	–
Spatio-temporal neural network (Chang et al., 2022)	(24, 29)	128	640 EEG features	Five-fold CV	71.14
Adaptive Channel Fusion-IFL (Shen et al., 2023)	(24,29)	128	space information	Five-fold CV	75.25
KNN (Cai et al., 2018a)	(92, 121)	3	270 features	Ten-fold CV	79.27
Convolutional Neural Network (Li et al., 2019)	(24, 27)	128	Spectral and Spatial Information	24-fold CV	85.62
Multimodal fusion (Zhang et al., 2019)	(81,89)	3 and voice(1)	6 EEG features and 15 voice features	Nested CV	86.64
Feature-level fusion (Cai et al., 2020)	(86, 92)	3	96 EEG features	Ten-fold CV	86.98
Improved Empirical Mode Decomposition (Shen et al., 2019)	(105, 70)	3	4	Ten-fold CV	88.07
SVM (Liu et al., 2020)	(20, 19)	64	3 potential biomarkers	Ten-fold CV	89.70
STARC (Ahmadlou et al., 2013)	(22, 20)	19	15,360	RRS	91.10
EEGNet (Liu et al., 2022)	(24, 29)	128	Preprocessed EEG	LOSOCV	90.98
Case-Based Reasoning Model (Cai et al., 2018b)	(86, 92)	3	113	Ten-fold CV	91.25
Fractality analysis (Ahmadlou et al., 2012)	(12, 12)	19	144	RRS	91.30
Multivariate pattern analysis (Peng et al., 2019)	(27, 28)	128	249	LOSOCV	92.73
Brain Function Networks (Zhang et al., 2020)	(24, 24)	64	LC-CC in theta band	Ten-fold CV	93.31
CFS-KNN (Li et al., 2018)	(19, 20)	14	1176 EEG features	LOSOCV	94.12
GainRatio-KNN (Li et al., 2018)	(19, 20)	14	1176	LOSOCV	94.12
Convolutional Neural Network (Acharya et al., 2018)	(15, 15)	4	Preprocessed EEG	Ten-fold CV	95.49
STARC (Ahmadlou et al., 2013)	(22, 20)	19	15,360	LOSOCV	95.80
Sequential Backward Feature Selection (Movahed et al., 2021)	(24, 29)	128	506 EEG features	Ten-fold CV	97.30
RWE-ANN (Puthankattil and Joseph, 2012)	(30, 30)	4	20	–	98.11
DCNN-LSTM (Saeedi et al., 2021)	(34, 30)	19	19×19 effective connectivity image	70% training, 30% testing	99.24
PC-STM-GM (Ours)	(21, 21)	EEG (128)	Preprocessed EEG features	LOPOCV	99.44*

Bold type highlights the result of the method proposed in this study. Results from different studies were obtained under different experimental protocols and are not directly comparable.

Within the present LOPOCV protocol, PC-STM-GM achieves 99.44% accuracy.

### 4.2 PD recognition results

**Figure 6** shows accuracy distributions across 24 subject pairs for the *PD ON vs. CTL* condition with 20 calibration trials. PC-STM-NP and PC-STM-GM outperform the Ensemble without STM configuration within this evaluation. While the baseline performance fluctuates between approximately 40% and 90%, PC-STM-GM remains high with limited variance.

**Figure 6:**
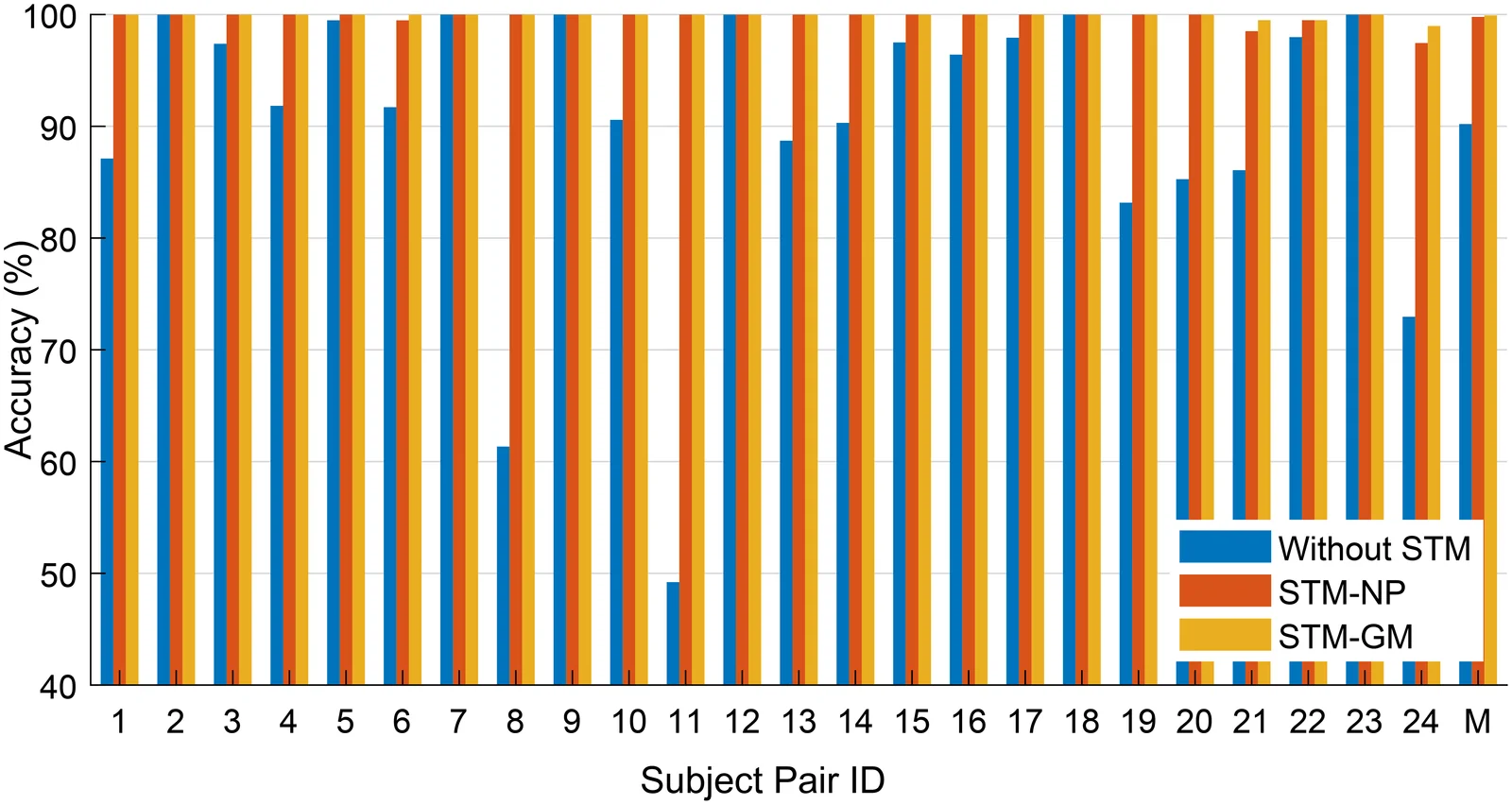
Recognition accuracy of the three methods for each pair of subjects in the PD ON vs. CTL experiment with 20 calibration trials. The label “M” on the x-axis denotes the mean accuracy across all 24 subjects.

To examine sensitivity to calibration size, **Figure 7** reports accuracy for the *PD ON vs. CTL* and *PD OFF vs. CTL* tasks using 5, 10, 15, and 20 calibration trials. The Ensemble without STM configuration is more sensitive to the number of calibration samples. In contrast, both PC-STM variants remain stable across the evaluated calibration sizes, and PC-STM-GM exceeds 99% in this transductive setting.

**Figure 7:**
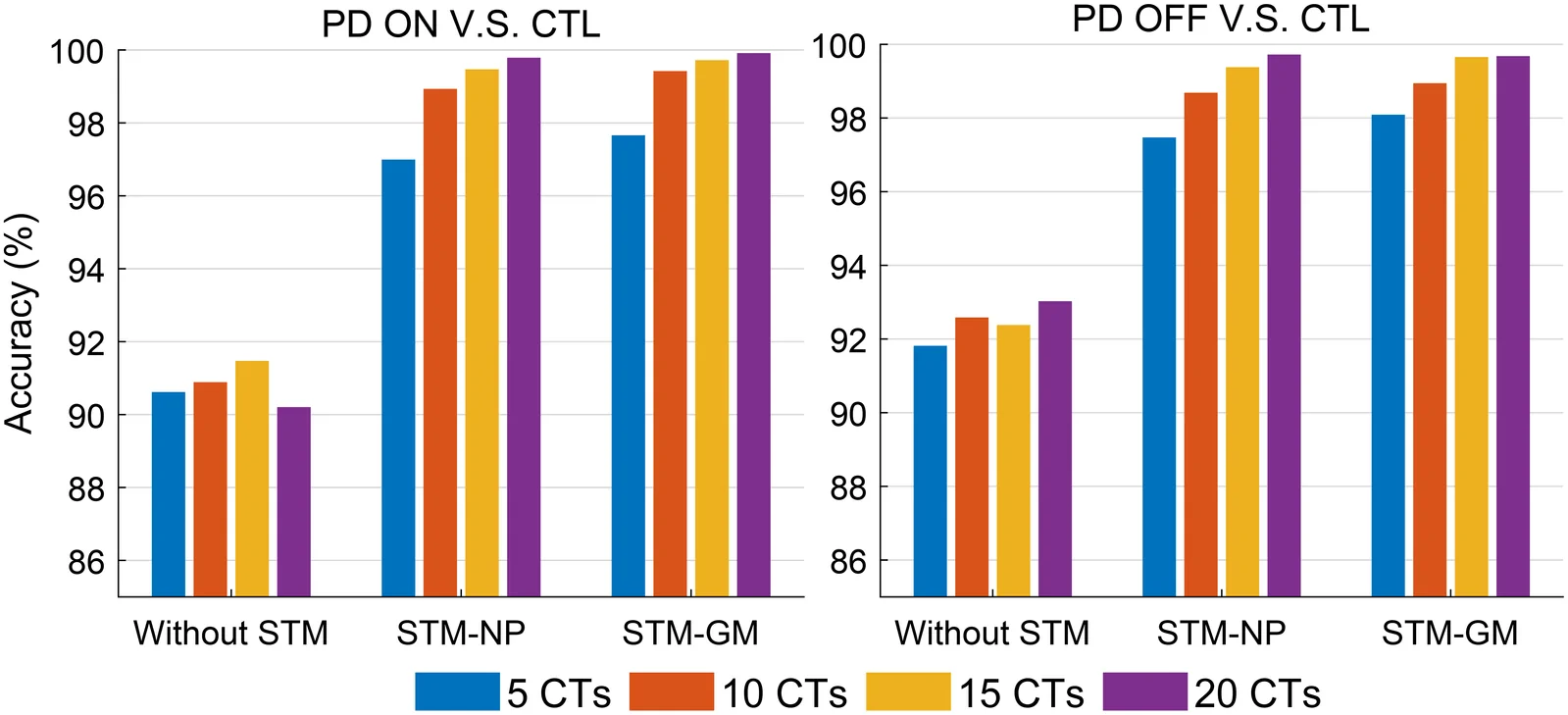
Recognition results of PD ON vs. CTL and PD OFF vs. CTL under different calibration sizes for the three methods. (CTs:calibration trails).

Confusion matrices in **Figure 8** further show the classification results. The baseline approach yields misclassification rates of 10.60% for CTL and 7.00% for PD. PC-STM-NP reduces these rates to 0.30% and 0.48%, respectively, while PC-STM-GM yields 0.13% and 0.22%, respectively. These findings show improved discrimination within the evaluated PD dataset.

**Figure 8:**
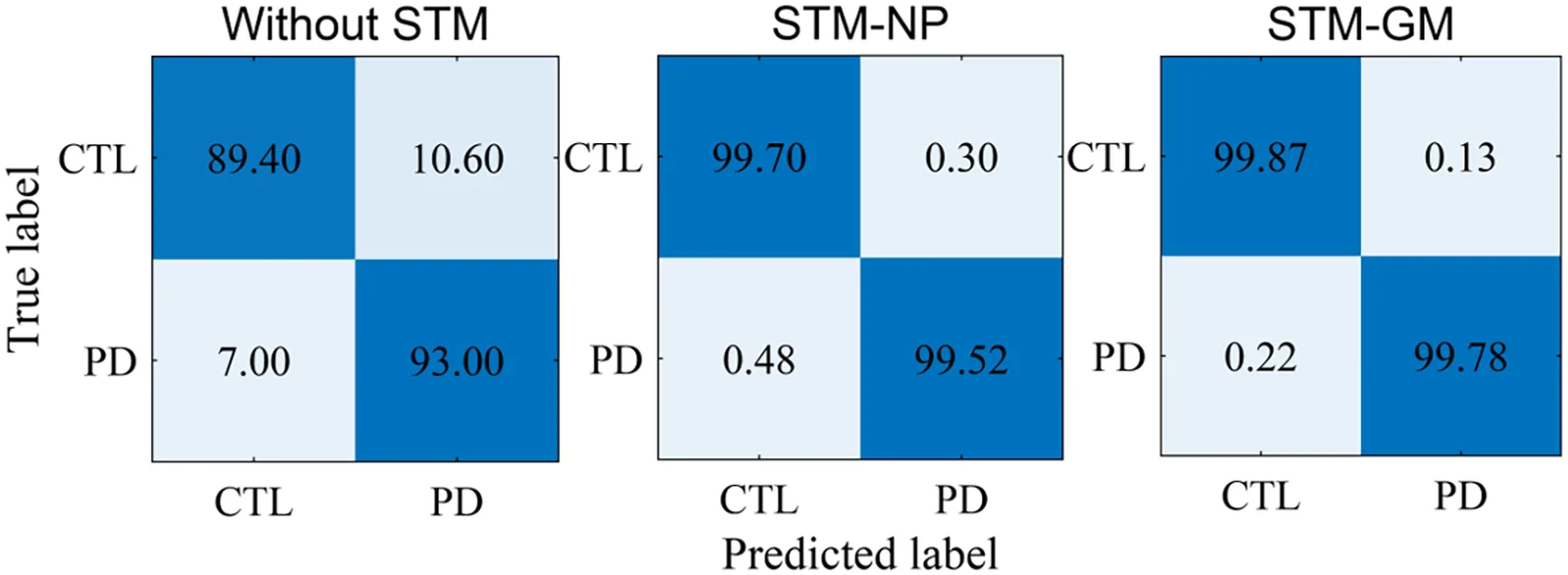
Confusion matrix of the three methods in the PD ON vs. CTL experiment with 20 calibration trials.

**Table 3** places PC-STM-GM alongside representative PD classification reports. The table is a contextual literature comparison rather than a controlled head-to-head evaluation because the studies differ in participants, EEG channels, preprocessing, features, and validation procedures. Within the present protocol, PC-STM-GM achieves 99.92% accuracy.

**Table 3:** Contextual comparison of PD recognition results reported under different experimental protocols.

Method	Subject (PD, CTL)	Channel	Accuracy (%)
Logistic Regression (Chaturvedi et al., 2017)	(50, 41)	214	56.00
J48 decision trees (Chaturvedi et al., 2017)	(50, 41)	214	68.00
SVM (Chaturvedi et al., 2017)	(50, 41)	214	74.70
Random Forest (Chaturvedi et al., 2017)	(50, 41)	214	78.00
CNN (Ruffini et al., 2019)	(14, 14)	1	79.00
RNN (Ruffini et al., 2019)	(14, 14)	1	81.00
SVM (Cavanagh et al., 2018)	(25, 25)	60	82.00
SVM (Betrouni et al., 2019)	(118, -)	122	86.00
KNN (Betrouni et al., 2019)	(118, -)	122	88.00
ASGCNN (Chang et al., 2023a)	(24, 24)	60	87.67
CNN (Oh et al., 2020)	(20, 20)	14	88.25
PC-STM-GM (Ours)	(24, 24)	60	99.92

The experimental results show that PC-STM improves classification stability relative to the same ensemble without STM. The Gaussian-model destination is more stable than the nearest-prototype destination in the reported experiments. These unusually high accuracies should be interpreted within the matched-pair design, public-dataset cohort labels, and transductive calibration protocol. Performance may decrease in independent cohorts, with different EEG devices or acquisition procedures, or without target calibration data.

## 5 Discussion

The experimental results show that PC-STM improves cross-subject EEG disease recognition within the two evaluated public datasets. This section discusses possible explanations for the observed results and states the limitations of the matched-pair transductive protocol.

### 5.1 Effectiveness of multi-source STM for reducing inter-subject variability

A major challenge in EEG-based disease recognition is the substantial variability in signal distribution across individuals. Direct training on source subjects often fails to generalize to unseen targets, as evidenced by the inconsistent performance of the baseline method. STM maps target samples toward class-wise prototypes constructed from multiple source subjects (Zhang and Liu, 2011; Zhang and Liu, 2012).

Compared with subject-level alignment or global distribution matching, prototype-guided STM provides more inspectable prototypes, affine parameters, and classifier weights, consistent with related transfer learning research (Yao and Doretto, 2010; Gu et al., 2022). This procedural transparency should not be interpreted as subject-level or neurophysiological explanation because the present study does not include saliency, source localization, or representation probing. The Nearest-Prototype (NP) method reduces coarse mismatches (Bezdek and Kuncheva, 2001), whereas the Gaussian-Model (GM) approach accounts for covariance geometry (Kimura et al., 1987). The observed accuracy pattern is consistent with smoother GM transformations, but it does not directly verify preservation of a physiological neural mechanism (Zubair et al., 2025).

### 5.2 Role of confidence-guided semi-supervised refinement

The protocol provides only a limited number of labeled calibration trials for a new target pair. These labels are inherited from the established dataset cohort or task condition rather than newly generated clinical trial-level diagnoses. Transductive STM uses the remaining evaluation samples through confidence-weighted pseudo-labels, while their ground-truth labels remain hidden. Samples with high certainty exert stronger influence on parameter updates, following the general principle of limiting uncertain supervision (Zhu et al., 2010; Zhang et al., 2025d). This mechanism is associated with the stability observed across the evaluated calibration sizes, but prospective deployment would require a realistic procedure for obtaining calibration labels.

### 5.3 Benefits of the performance-weighted ensemble strategy

The ensemble framework complements STM by integrating discriminative characteristics from multiple subject pairs, following the principle of SVM classifier fusion (Ceamanos et al., 2010). Sub-classifiers with higher calibration accuracy receive higher weights, reflecting their measured relevance to the target pair. This weighting is a statistical procedure and is not intended to reproduce clinical reasoning.

The combination of STM and ensemble learning significantly improves robustness, reducing reliance on any single source subject. This property is particularly beneficial when the target subject exhibits unique EEG patterns not well represented in the training set.

### 5.4 Evidence boundaries and potential extensions

The proposed framework performs consistently in the evaluated MDD and PD datasets, which differ in task design and EEG characteristics (Chang et al., 2023b; Cui et al., 2023). This classification stability is consistent with reduced subject-related feature mismatch while retaining disease-group discrimination. It is an interpretation of predictive behavior rather than direct evidence that STM physiologically isolates subject-specific variation or preserves condition-specific neural signatures.

Application to anxiety, bipolar disorder, cognitive impairment, or non-EEG modalities remains a potential extension and has not been demonstrated in this study. Such applications require independent data, task-specific validation, and prospective evaluation. Cross-domain EEG generalization remains challenging in other settings, including driver-state detection (Ju and Li, 2024).

Future research may evaluate nonlinear STM formulations, adaptive prototype learning, multimodal physiological inputs, independent cohorts, device shifts, and calibration-free inference (Schlett et al., 2023; Nguyen et al., 2024). Generalization-preserved learning in other continual-recognition settings also reinforces the need for explicit stress tests (Zhang et al., 2025e). At present, PC-STM should be viewed as a transparent recognition framework under a controlled retrospective protocol rather than validated clinical decision support (Jain et al., 2021).

## 6 Conclusion

This study introduces PC-STM with a calibration-informed weighted ensemble for cross-subject EEG-based neuropsychiatric disease recognition. Within the evaluated matched-pair transductive protocol, PC-STM improves classification stability relative to the same ensemble without STM, and PC-STM-GM provides the highest accuracy in the MDD and PD datasets. These findings do not establish prospective diagnostic utility, calibration-free performance, physiological interpretability, or generalization to other disorders and devices. Future work should evaluate independent cohorts, calibration-free settings, cross-device transfer, and prospective recognition workflows before clinical application is considered.

## Data Availability

The original contributions presented in the study are included in the article/**Supplementary Material**. Further inquiries can be directed to the corresponding author.

## Footnotes

1
http://modma.lzu.edu.cn/data/application/#data1

2
https://sccn.ucsd.edu/eeglab/index.php

3
http://predict.cs.unm.edu/downloads.php
