# Supplementary material for: Style transfer mapping with ensemble learning for cross-subject EEG-based neuropsychiatric disease recognition
Source: Front Physiol. 2026 Oct 14;17:1947840. doi: 10.3389/fphys.2026.1947840 (PMC13713322; doi:10.3389/fphys.2026.1947840)
Supplement: Supplementary file 1 [file DataSheet1.pdf]

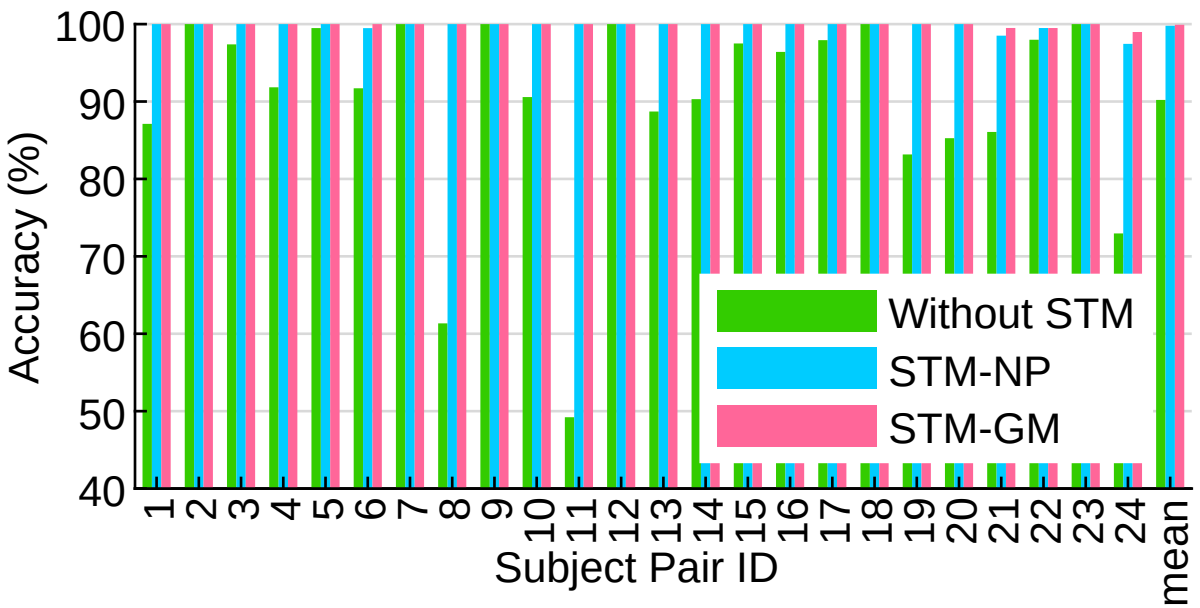

Accuracy (%)
100
90
80
70
60
50
40
1
2
3
4
5
6
7
8
9
10
11
12
13
14
15
16
17
18
19
20
21
22
23
24
mean
Subject Pair ID
Without STM
STM-NP
STM-GM
