# Supplementary material for: Style transfer mapping with ensemble learning for cross-subject EEG-based neuropsychiatric disease recognition
Source: Front Physiol. 2026 Oct 14;17:1947840. doi: 10.3389/fphys.2026.1947840 (PMC13713322; doi:10.3389/fphys.2026.1947840)
Supplement: Supplementary file 2 [file DataSheet10.pdf]

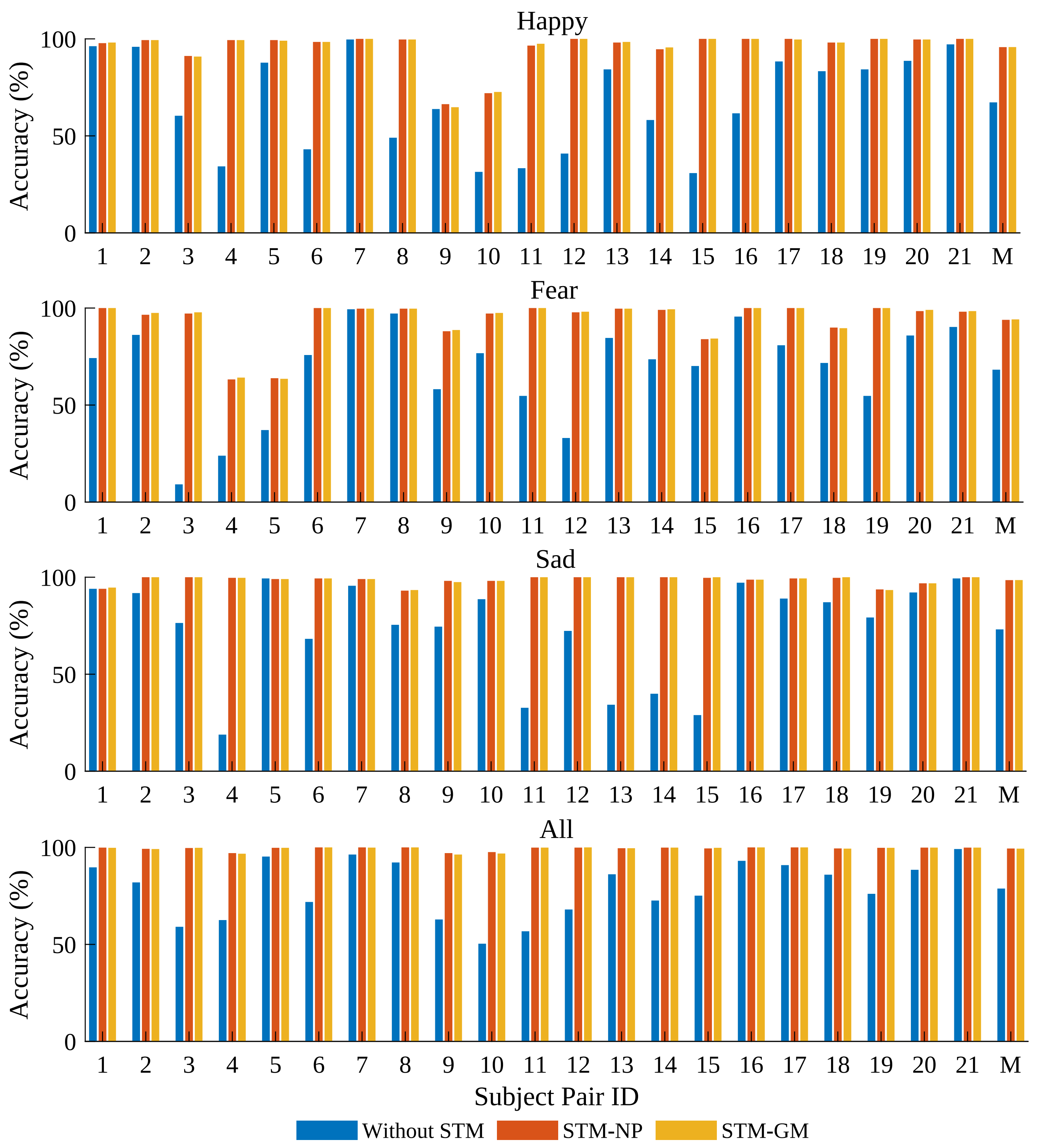
Happy
Fear
Sad
All
Accuracy (%)
0
50
100
1 2 3 4 5 6 7 8 9 10 11 12 13 14 15 16 17 18 19 20 21 M
Subject Pair ID
Without STM
STM-NP
STM-GM
