# Supplementary material for: Style transfer mapping with ensemble learning for cross-subject EEG-based neuropsychiatric disease recognition
Source: Front Physiol. 2026 Oct 14;17:1947840. doi: 10.3389/fphys.2026.1947840 (PMC13713322; doi:10.3389/fphys.2026.1947840)
Supplement: Supplementary file 3 [file DataSheet11.pdf]

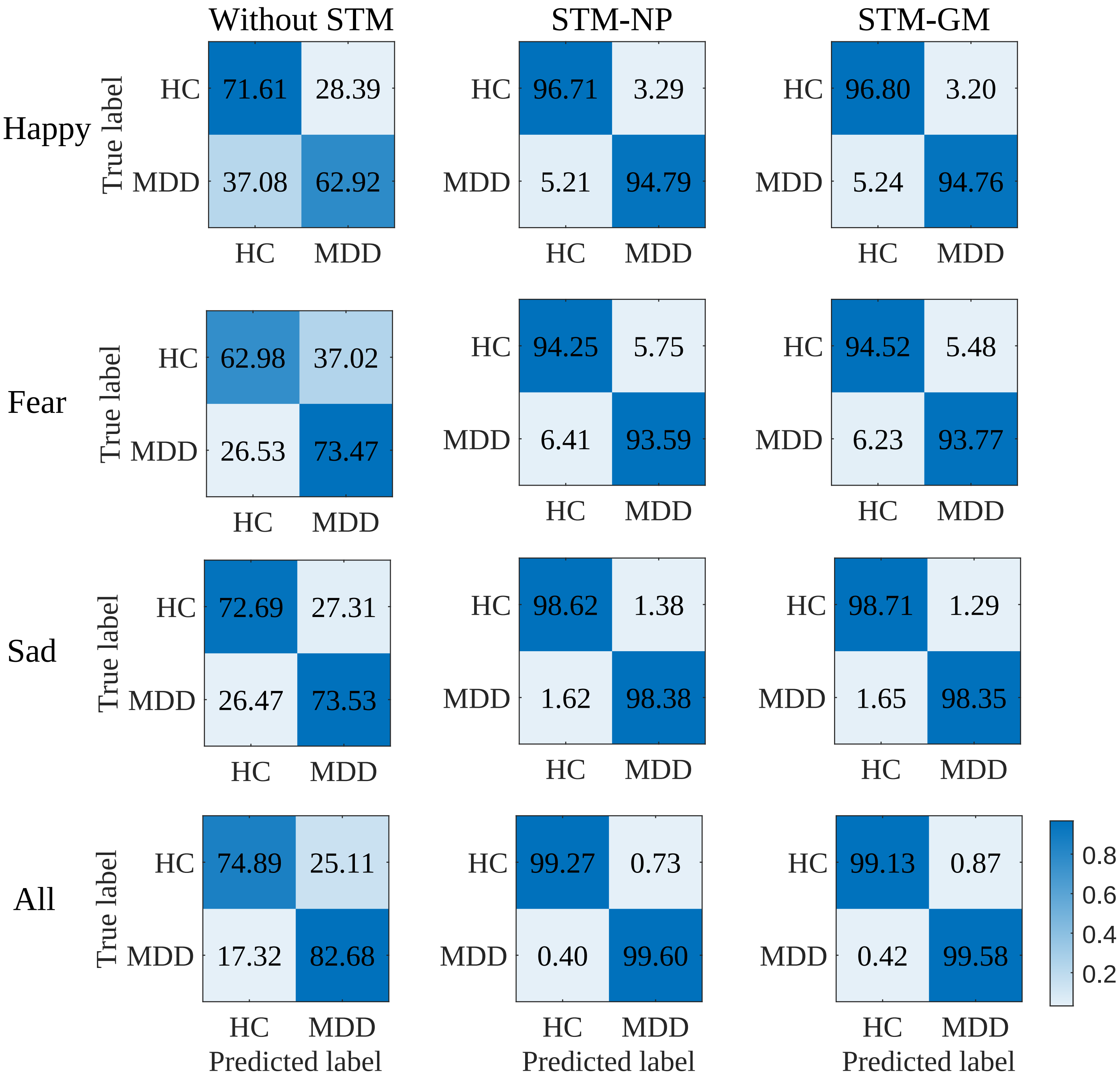

Without STM
STM-NP
STM-GM
Happy
Fear
Sad
All
True label
Predicted label
HC
MDD
71.61
28.39
37.08
62.92
96.71
3.29
5.21
94.79
96.80
3.20
5.24
94.76
62.98
37.02
26.53
73.47
94.25
5.75
6.41
93.59
94.52
5.48
6.23
93.77
72.69
27.31
26.47
73.53
98.62
1.38
1.62
98.38
98.71
1.29
1.65
98.35
74.89
25.11
17.32
82.68
99.27
0.73
0.40
99.60
99.13
0.87
0.42
99.58
0.8
0.6
0.4
0.2
