# Supplementary material for: Style transfer mapping with ensemble learning for cross-subject EEG-based neuropsychiatric disease recognition
Source: Front Physiol. 2026 Oct 14;17:1947840. doi: 10.3389/fphys.2026.1947840 (PMC13713322; doi:10.3389/fphys.2026.1947840)
Supplement: Supplementary file 4 [file DataSheet12.pdf]

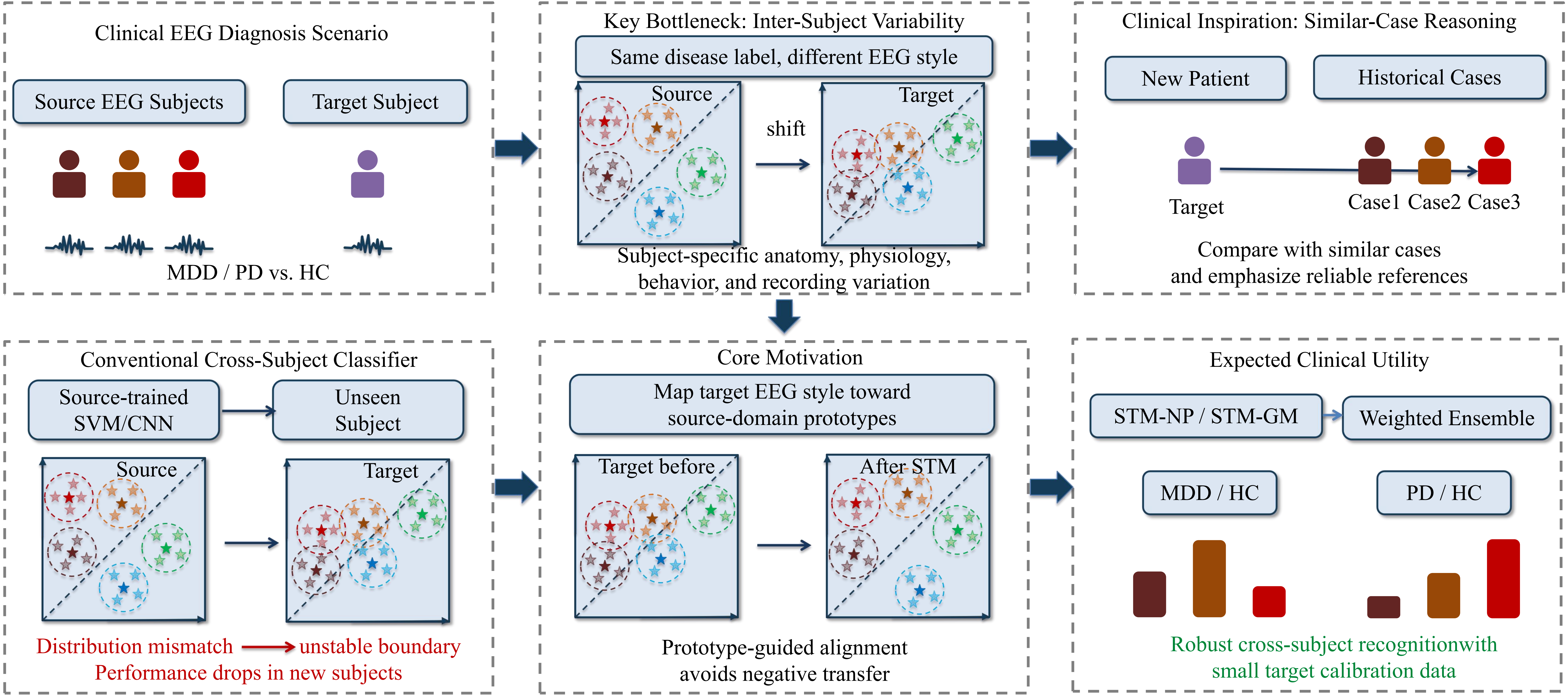

Clinical EEG Diagnosis Scenario
Source EEG Subjects
Target Subject
MDD / PD vs. HC
Key Bottleneck: Inter-Subject Variability
Same disease label, different EEG style
Source
shift
Target
Subject-specific anatomy, physiology, behavior, and recording variation
Clinical Inspiration: Similar-Case Reasoning
New Patient
Historical Cases
Target
Case1
Case2
Case3
Compare with similar cases and emphasize reliable references
Conventional Cross-Subject Classifier
Source-trained SVM/CNN
Unseen Subject
Source
Target
Distribution mismatch
unstable boundary
Performance drops in new subjects
Core Motivation
Map target EEG style toward source-domain prototypes
Target before
After STM
Prototype-guided alignment avoids negative transfer
Expected Clinical Utility
STM-NP / STM-GM
Weighted Ensemble
MDD / HC
PD / HC
Robust cross-subject recognitionwith small target calibration data
