# Supplementary material for: Style transfer mapping with ensemble learning for cross-subject EEG-based neuropsychiatric disease recognition
Source: Front Physiol. 2026 Oct 14;17:1947840. doi: 10.3389/fphys.2026.1947840 (PMC13713322; doi:10.3389/fphys.2026.1947840)
Supplement: Supplementary file 5 [file DataSheet13.pdf]

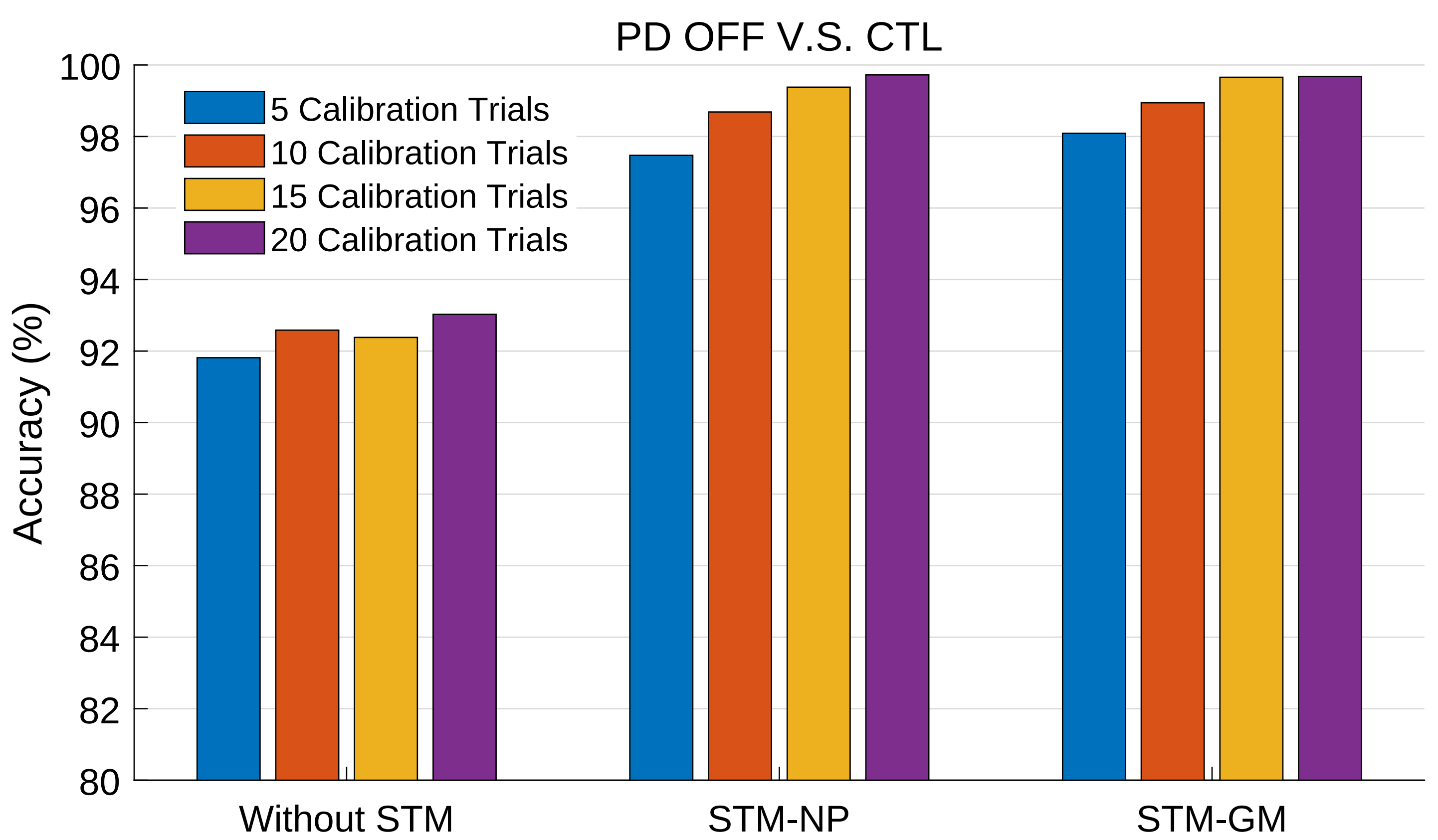

PD OFF V.S. CTL
Accuracy (%)
100
98
96
94
92
90
88
86
84
82
80
5 Calibration Trials
10 Calibration Trials
15 Calibration Trials
20 Calibration Trials
Without STM
STM-NP
STM-GM
