# Supplementary material for: Style transfer mapping with ensemble learning for cross-subject EEG-based neuropsychiatric disease recognition
Source: Front Physiol. 2026 Oct 14;17:1947840. doi: 10.3389/fphys.2026.1947840 (PMC13713322; doi:10.3389/fphys.2026.1947840)
Supplement: Supplementary file 9 [file DataSheet3.pdf]

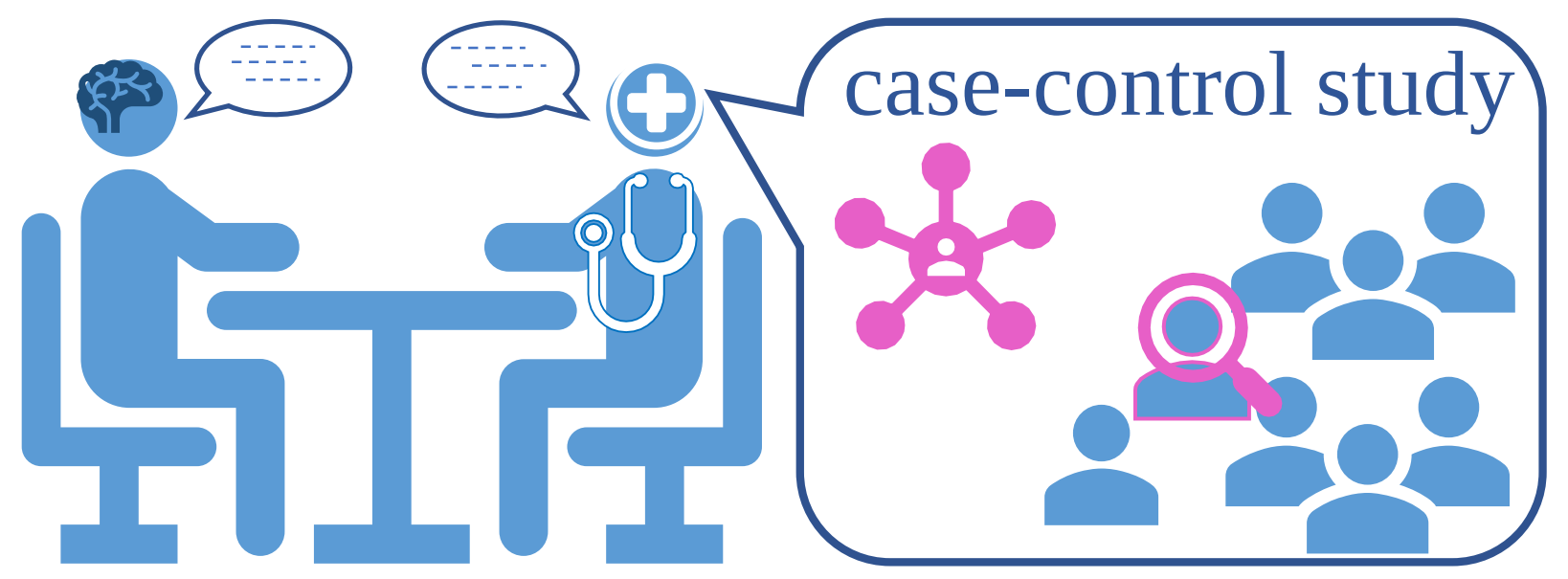
case-control study
