# Supplementary material for: Style transfer mapping with ensemble learning for cross-subject EEG-based neuropsychiatric disease recognition
Source: Front Physiol. 2026 Oct 14;17:1947840. doi: 10.3389/fphys.2026.1947840 (PMC13713322; doi:10.3389/fphys.2026.1947840)
Supplement: Supplementary file 11 [file DataSheet5.pdf]

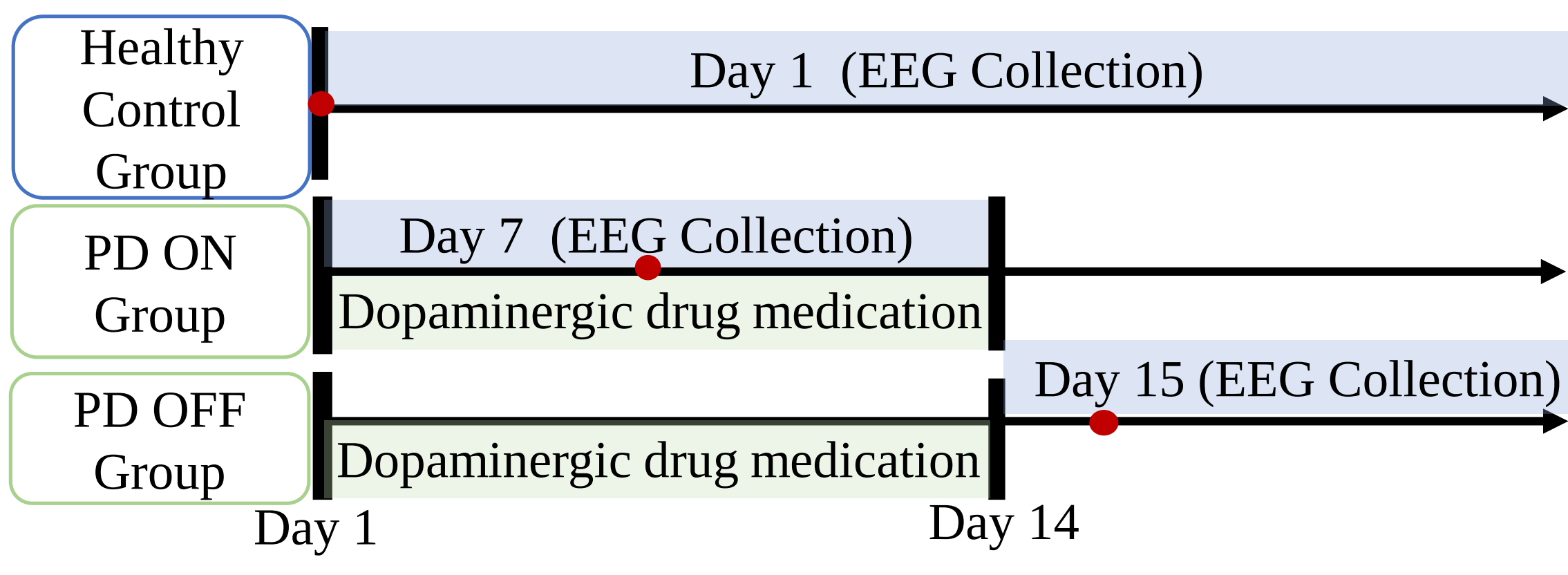
Healthy Control Group
Day 1 (EEG Collection)
PD ON Group
Day 7 (EEG Collection)
Dopaminergic drug medication
Day 15 (EEG Collection)
PD OFF Group
Dopaminergic drug medication
Day 1
Day 14
