# Supplementary material for: Style transfer mapping with ensemble learning for cross-subject EEG-based neuropsychiatric disease recognition
Source: Front Physiol. 2026 Oct 14;17:1947840. doi: 10.3389/fphys.2026.1947840 (PMC13713322; doi:10.3389/fphys.2026.1947840)
Supplement: Supplementary file 12 [file DataSheet6.pdf]

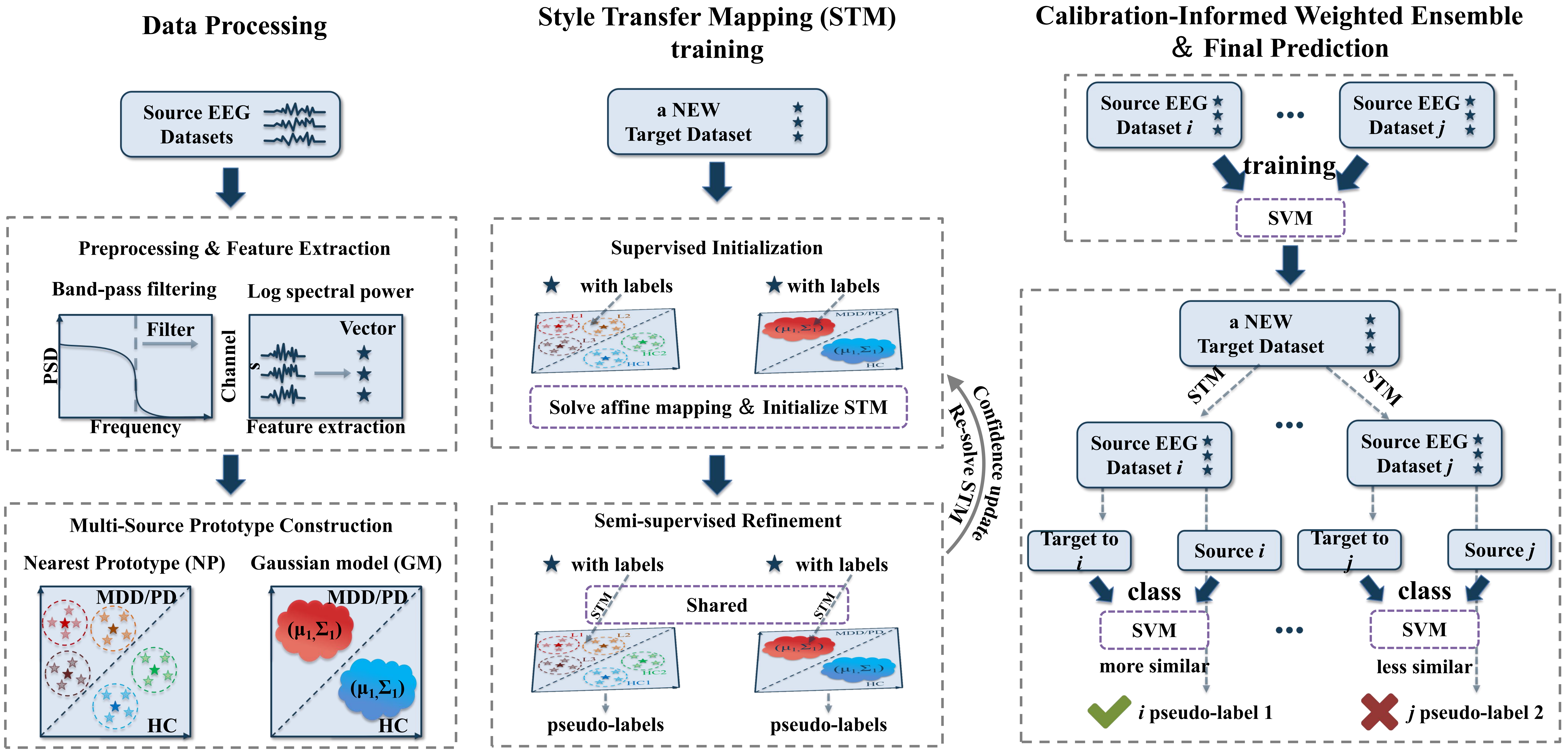
Data Processing
Source EEG Datasets
Preprocessing & Feature Extraction
Band-pass filtering
Log spectral power
Filter
PSD
Frequency
Channels
Vector
Feature extraction
Multi-Source Prototype Construction
Nearest Prototype (NP)
Gaussian model (GM)
MDD/PD
HC
(μ1,Σ1)
Style Transfer Mapping (STM) training
a NEW Target Dataset
Supervised Initialization
with labels
with labels
Solve affine mapping & Initialize STM
Semi-supervised Refinement
with labels
with labels
Shared
STM
STM
pseudo-labels
pseudo-labels
Confidence update
Re-solve STM
Calibration-Informed Weighted Ensemble & Final Prediction
Source EEG Dataset i
Source EEG Dataset j
training
SVM
a NEW Target Dataset
STM
STM
Source EEG Dataset i
Source EEG Dataset j
Target to i
Source i
Target to j
Source j
class
class
SVM
SVM
more similar
less similar
i pseudo-label 1
j pseudo-label 2
