# Supplementary material for: Style transfer mapping with ensemble learning for cross-subject EEG-based neuropsychiatric disease recognition
Source: Front Physiol. 2026 Oct 14;17:1947840. doi: 10.3389/fphys.2026.1947840 (PMC13713322; doi:10.3389/fphys.2026.1947840)
Supplement: Supplementary file 13 [file DataSheet7.pdf]

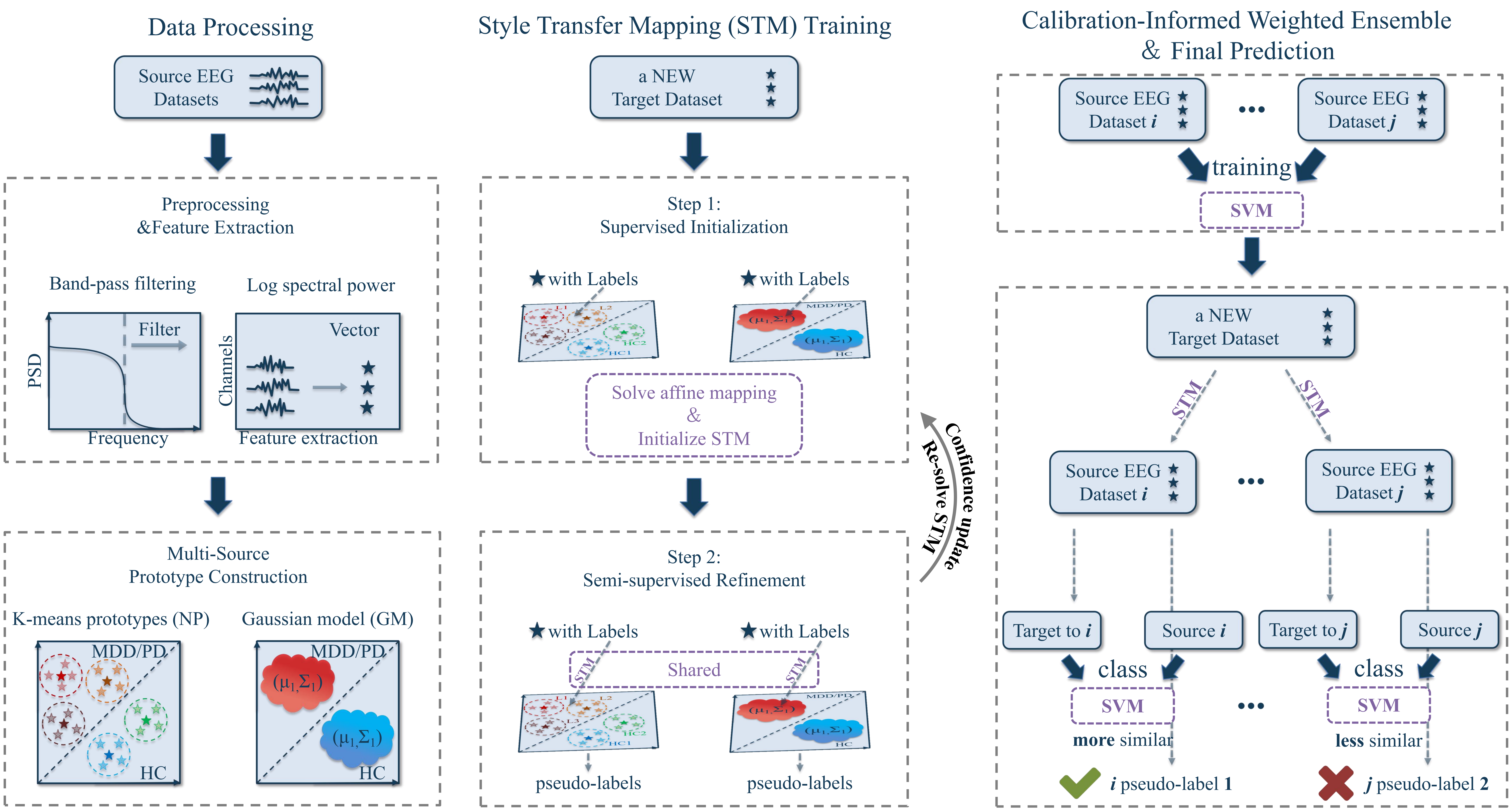

Data Processing
Source EEG Datasets
Preprocessing &Feature Extraction
Band-pass filtering
Log spectral power
Filter
PSD
Frequency
Vector
Channels
Feature extraction
Multi-Source Prototype Construction
K-means prototypes (NP)
Gaussian model (GM)
MDD/PD
HC
Style Transfer Mapping (STM) Training
a NEW Target Dataset
Step 1: Supervised Initialization
with Labels
Solve affine mapping & Initialize STM
Step 2: Semi-supervised Refinement
Shared
STM
pseudo-labels
Confidence update
Re-solve STM
Calibration-Informed Weighted Ensemble & Final Prediction
Source EEG Dataset i
Source EEG Dataset j
training
SVM
Target to i
Source i
Target to j
Source j
class
more similar
less similar
i pseudo-label 1
j pseudo-label 2

EEG Datasets
(MDD / PD)

Preprocessing & Feature Extraction
- Band-pass filtering
- Log spectral power

Multi-Source Prototype Construction
- K-means prototypes (NP)
- Gaussian model (GM)

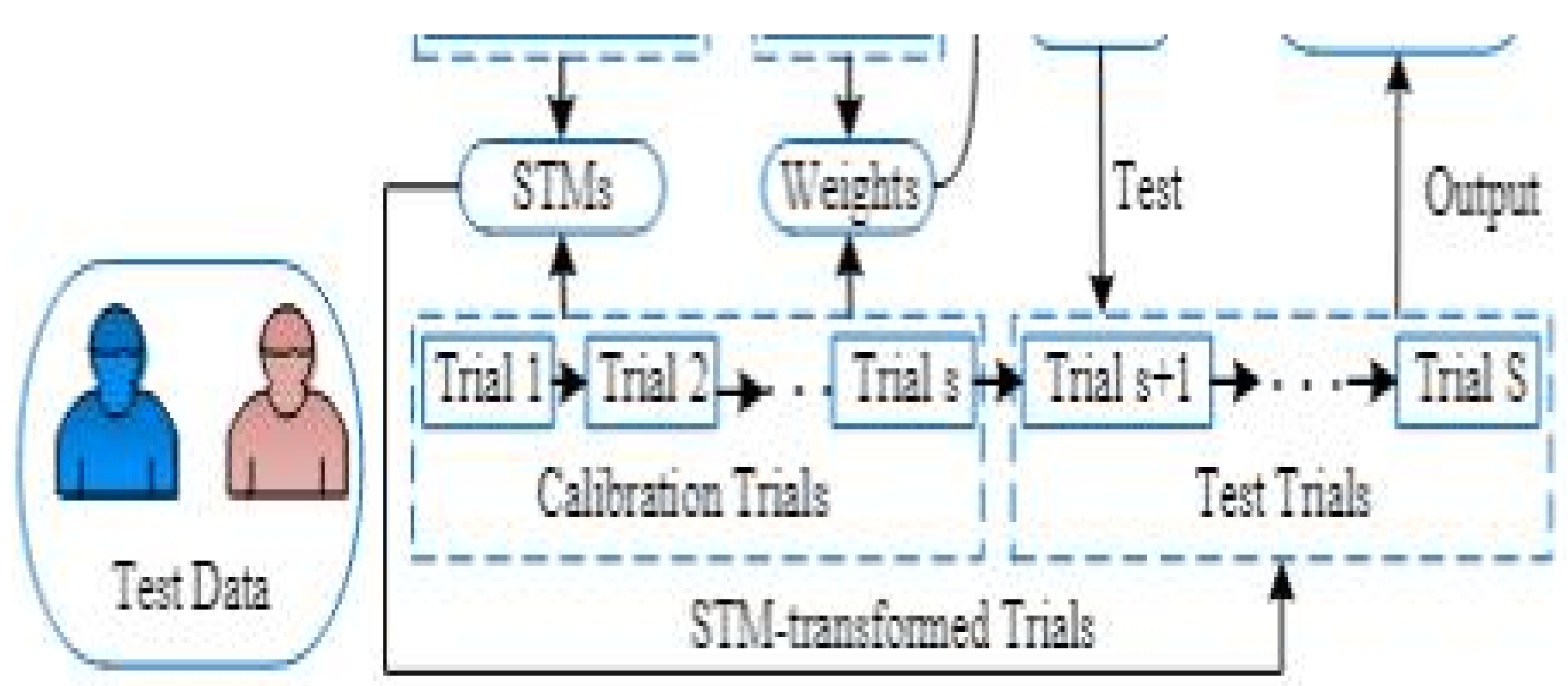

Style Transfer Mapping (STM)
Supervised Initialization:
- Assign destinations (NP/GM)
- Solve affine mapping

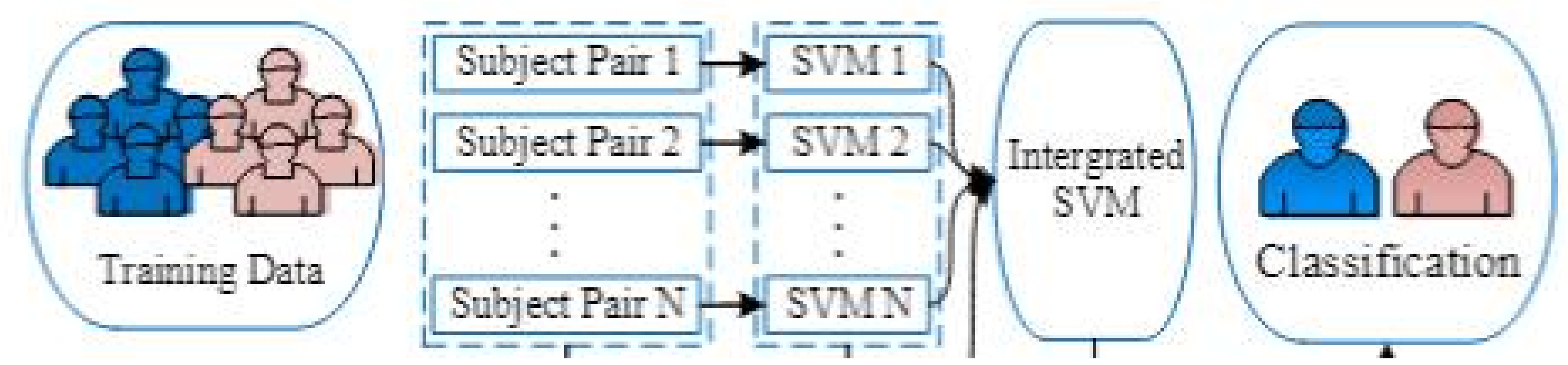

Calibration-Informed Weighted Ensemble
- Train SVMs on source pairs
- Compute weights
- Final decision

Semi-supervised Refinement:
- Predict pseudo-labels
- Confidence update
- Re-solve STM

Final Prediction
(MDD / PD)
