# Supplementary material for: Style transfer mapping with ensemble learning for cross-subject EEG-based neuropsychiatric disease recognition
Source: Front Physiol. 2026 Oct 14;17:1947840. doi: 10.3389/fphys.2026.1947840 (PMC13713322; doi:10.3389/fphys.2026.1947840)
Supplement: Supplementary file 14 [file DataSheet8.pdf]

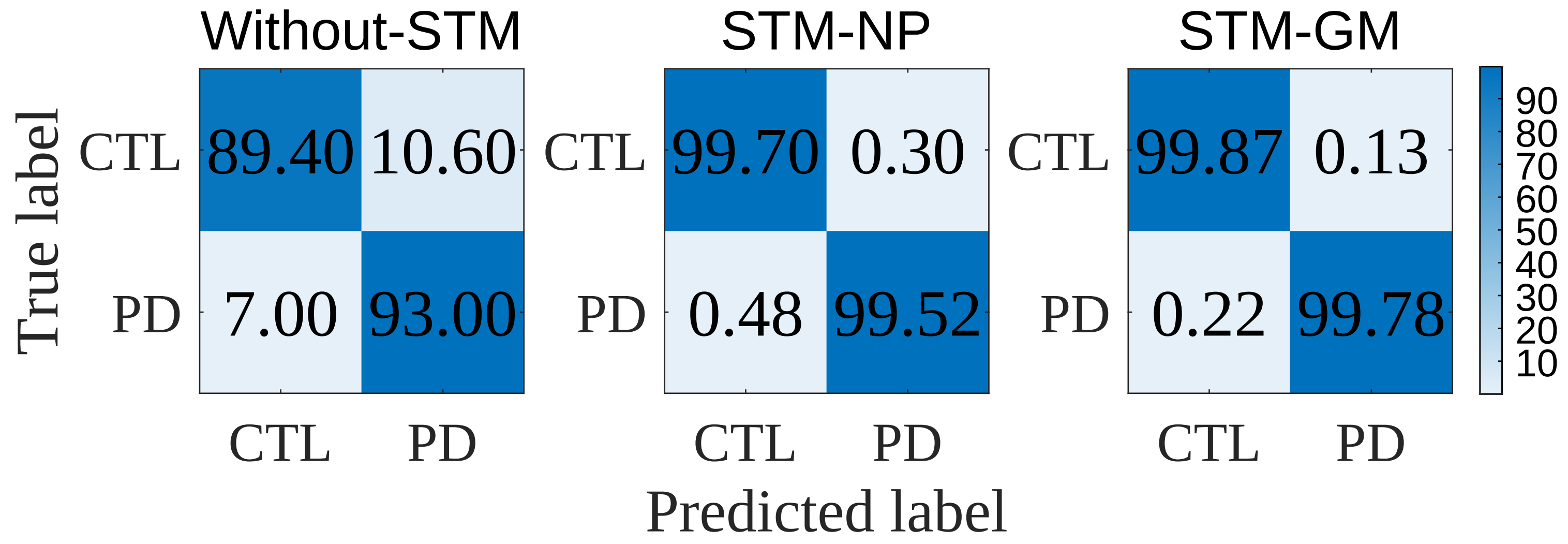
Without-STM
STM-NP
STM-GM
True label
Predicted label
CTL
PD
89.40
10.60
7.00
93.00
99.70
0.30
0.48
99.52
99.87
0.13
0.22
99.78
10
20
30
40
50
60
70
80
90
