# Supplementary material for: Style transfer mapping with ensemble learning for cross-subject EEG-based neuropsychiatric disease recognition
Source: Front Physiol. 2026 Oct 14;17:1947840. doi: 10.3389/fphys.2026.1947840 (PMC13713322; doi:10.3389/fphys.2026.1947840)
Supplement: Supplementary file 15 [file DataSheet9.pdf]

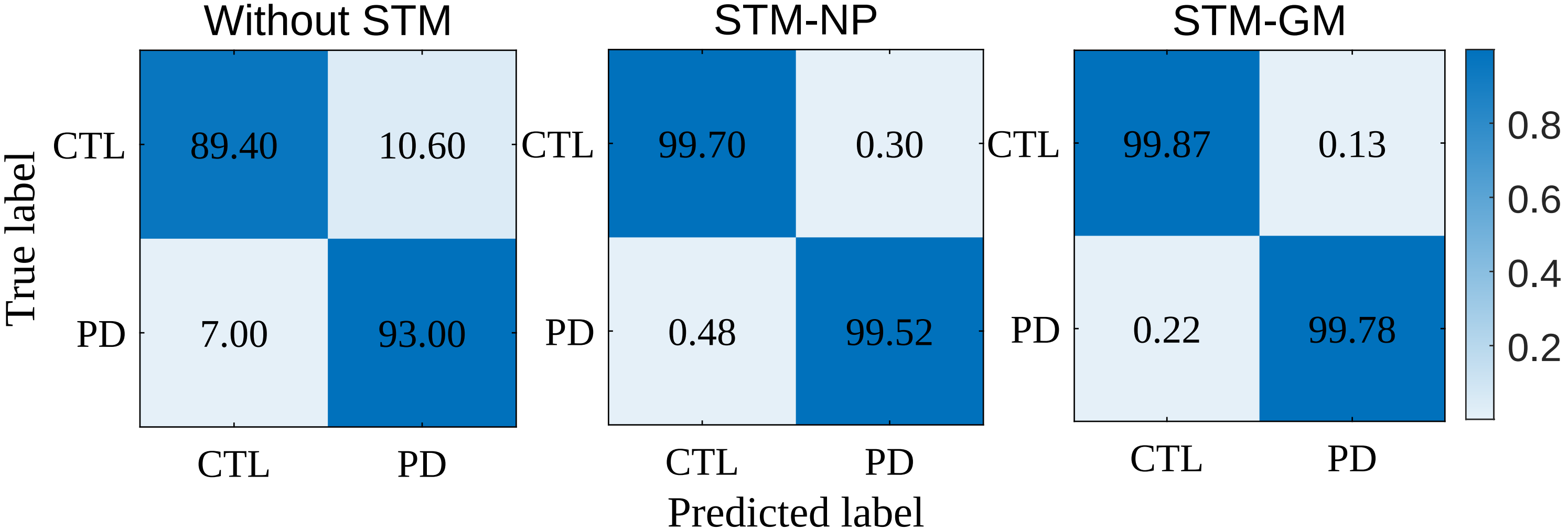

Without STM
STM-NP
STM-GM
True label
CTL
PD
89.40
10.60
7.00
93.00
99.70
0.30
0.48
99.52
99.87
0.13
0.22
99.78
0.8
0.6
0.4
0.2
Predicted label
